# A Plant Gene Encoding One-Heme and Two-Heme Hemoglobins With Extreme Reactivities Toward Diatomic Gases and Nitrite

**DOI:** 10.3389/fpls.2020.600336

**Published:** 2020-11-19

**Authors:** Irene Villar, Estíbaliz Larrainzar, Lisa Milazzo, Carmen Pérez-Rontomé, Maria C. Rubio, Giulietta Smulevich, Jesús I. Martínez, Michael T. Wilson, Brandon Reeder, Raul Huertas, Stefania Abbruzzetti, Michael Udvardi, Manuel Becana

**Affiliations:** ^1^Departamento de Nutrición Vegetal, Estación Experimental de Aula Dei, Consejo Superior de Investigaciones Científicas (CSIC), Zaragoza, Spain; ^2^Department of Sciences, Institute for Multidisciplinary Applied Biology, Campus Arrosadía, Universidad Pública de Navarra, Pamplona, Spain; ^3^Dipartimento di Chimica "Ugo Schiff", Università di Firenze, Florence, Italy; ^4^Instituto de Ciencia de Materiales de Aragón, Universidad de Zaragoza-CSIC, Zaragoza, Spain; ^5^School of Life Sciences, Essex University, Wivenhoe Park, Colchester, United Kingdom; ^6^Noble Research Institute LLC, Ardmore, OK, United States; ^7^Dipartimento di Scienze Matematiche, Fisiche e Informatiche, Università di Parma, Parma, Italy

**Keywords:** *Medicago truncatula*, nitric oxide, symbiosis, phytoglobins, nodule, leghemoglobin, nitrate, hypoxia

## Abstract

In plants, symbiotic hemoglobins act as carriers and buffers of O_2_ in nodules, whereas nonsymbiotic hemoglobins or phytoglobins (Glbs) are ubiquitous in tissues and may perform multiple, but still poorly defined, functions related to O_2_ and/or nitric oxide (NO). Here, we have identified a *Glb* gene of the model legume *Medicago truncatula* with unique properties. The gene, designated *MtGlb1-2*, generates four alternative splice forms encoding Glbs with one or two heme domains and 215–351 amino acid residues. This is more than double the size of any hemoglobin from plants or other organisms described so far. A combination of molecular, cellular, biochemical, and biophysical methods was used to characterize these novel proteins. RNA-sequencing showed that the four splice variants are expressed in plant tissues. *MtGlb1-2* is transcriptionally activated by hypoxia and its expression is further enhanced by an NO source. The gene is preferentially expressed in the meristems and vascular bundles of roots and nodules. Two of the proteins, bearing one or two hemes, were characterized using mutants in the distal histidines of the hemes. The Glbs are extremely reactive toward the physiological ligands O_2_, NO, and nitrite. They show very high O_2_ affinities, NO dioxygenase activity (in the presence of O_2_), and nitrite reductase (NiR) activity (in the absence of O_2_) compared with the hemoglobins from vertebrates and other plants. We propose that these Glbs act as either NO scavengers or NO producers depending on the O_2_ tension in the plant tissue, being involved in the fast and fine tuning of NO concentration in the cytosol in response to sudden changes in O_2_ availability.

## Introduction

Hemoglobins are ubiquitously present in the archaea, bacteria, and eukaryotes (Vinogradov et al., 2005). They typically comprise a heme prosthetic group and a polypeptide of 6–8 α-helices. The heme is an iron (Fe)-protoporphyrin IX with the ability to bind diatomic gases of biological relevance such as molecular oxygen (O_2_) and nitric oxide (NO). Binding of O_2_ occurs exclusively with the Fe^2+^ of the heme, whereas NO can bind Fe^2+^ with high affinity and Fe^3+^ with low affinity. Hemoglobins can be classified structurally according to the axial coordination of the heme Fe. Pentacoordinate hemoglobins have a His residue at the fifth position (proximal) leaving the sixth position (distal) to be occupied by water or gaseous ligands, whereas hexacoordinate hemoglobins have a second His, or much less frequently Lys, Gln, or Tyr, at the sixth position (Becana et al., 2020).

In plants, there are two major types of hemoglobins. Symbiotic hemoglobins occur in the root nodules of legumes, *Parasponia*, and actinorhizal plants, and their function is to deliver a low but steady concentration of O_2_ to the microbial partners, thus preventing nitrogenase inactivation (Appleby, 1984; Tjepkema et al., 1986) The symbiotic hemoglobins of legumes (leghemoglobins; Lbs) are found at concentrations of 1–5 mM in nodules and are pentacoordinate (Smagghe et al., 2009). In contrast, the nonsymbiotic hemoglobins or phytoglobins (Glbs) are expressed in virtually all plant tissues, with estimated concentrations of 5–12 μM (Duff et al., 1998). They can be categorized into three phylogenetic classes. Class 1 Glbs have a very high O_2_ affinity and hence probably do not act as O_2_ transporters. At least some of their functions may be related to the plant’s tolerance to hypoxia and NO homeostasis (Igamberdiev and Hill, 2004). Class 2 Glbs have homology with Lbs and show moderate O_2_ affinity, which is compatible with a role in modulation of O_2_ and/or NO levels (Smagghe et al., 2009). Class 3 or “truncated” Glbs have low O_2_ affinity and unknown functions. Class 1 and 2 Glbs are hexacoordinate but class 3 Glbs may adopt any of the two coordination states (Watts et al., 2001; Smagghe et al., 2009)

Legumes contain a variable number of Lbs and Glbs as exemplified by the model species *Medicago truncatula* and *Lotus japonicus*, commonly used for genetic and molecular studies (Berger et al., 2020b; Larrainzar et al., 2020). We have identified a *Glb* gene of *M. truncatula*, Medtr4g068870, designated as *MtGlb1-2*, with unique properties. *In silico* analysis predicts that it generates four alternative splice forms encoding Glbs with one or two heme domains and 215–351 amino acid residues. This size may double the range of 145–170 amino acids observed so far in plant hemoglobins (Berger et al., 2020b; Larrainzar et al., 2020). To date, this Glb gene and the derived proteins have not been examined in detail in any other plant or organism, which led us to undertake their functional characterization using a multidisciplinary approach. Our results show that the different splice forms, including those encoding the long proteins with two heme groups, are expressed in roots and nodules. Furthermore, we show that the gene is transcriptionally activated by hypoxia and NO in roots, and its expression localized in the meristems and vascular bundles of roots and nodules. The gene is transcriptionally activated by hypoxia and NO and is expressed in the meristems and vascular bundles of roots and nodules. The proteins are extremely reactive toward diatomic gases and nitrite (NO_2_^−^) and show very high NO dioxygenase (NOD) and nitrite reductase (NiR) activities. We propose that these Glbs act as either NO scavengers or NO producers depending on the O_2_ tension in the plant tissue and that they are involved in the fast and fine tuning of NO concentration in the cytosol in response to rapid changes in O_2_ availability.

## Materials and Methods

### Plant Growth and Expression Analyses of *MtGlb1-2*

Seeds of *M. truncatula* Jemalong A17 were scarified, sterilized, imbibed for 8 h at 25°C, synchronized for 2 days at 4°C on 0.5% (w/v) agar plates, and germinated at 23/21°C for 1 day in the dark and 2 more days with a 16-h photoperiod. After germination, seedlings were transferred to Fahräeus plates and grown for 11 days in controlled-environment cabinets with a day/night regime of 23/21°C, 200 μmol m^−2^ s^−1^ and 16-h photoperiod. Two sets of experiments were performed. In the first one, plants were transferred to fresh plates containing either unsupplemented medium or medium supplemented with 2 mM NH_4_Cl or 2 mM KNO_3_. Plants were harvested at 24 or 48 h after replating. In the second experiment, roots were submerged in 5 mM phosphate buffer (pH 7.0) alone (hypoxia) or in buffer supplemented with 2 mM NH_4_Cl, 2 mM KNO_3_, or 2 mM GSNO for 24 or 48 h. Roots were harvested in liquid N_2_ and stored at −80°C until their use.

For RNA-sequencing (RNA-seq) analyses of the *MtGlb1-2* splice forms *in vivo*, the different plant organs were isolated from plants grown in cones (Stuewue & Sons, Tangent, Oregon, United States) containing a 2:1 ratio of Turface to vermiculite. Five-week-old plants were used to sample roots and leaves, while 8‐ to 10-week-old plants were used to sample small (immature seeds), medium (green), and large (yellow) pods. For the nodulation time points, plants were grown in aeroponic chambers and were inoculated with *Sinorhizobium medicae* strain ABS7 as previously described (de Bang et al., 2017). Nodules were harvested from 4 to 28 days post-inoculation (dpi). At 14 dpi, a subset of plants were treated with 10 mM KNO_3_ for 12 and 48 h. All experiments were performed with three biological replicates. All harvested material was frozen immediately in liquid nitrogen and stored at −80°C prior to RNA isolation. RNA-seq library preparation, transcriptome sequencing, and mapping were performed according to de Bang et al. (2017).

**Figure 1:**
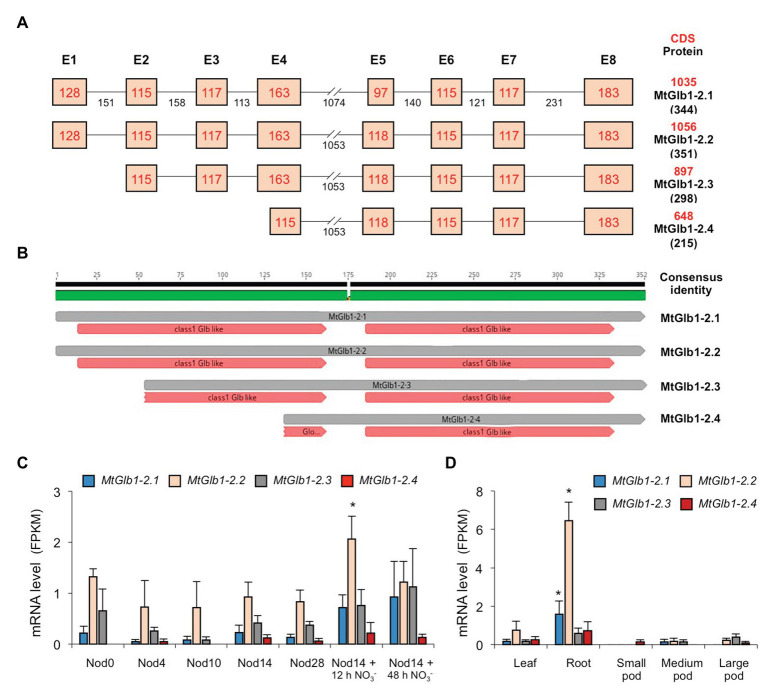
Structure and expression of the *MtGlb1-2* (Medtr4g068870) gene. **(A)** Exon and intron composition of the four alternative splice forms (*MtGlb1-2.1* to *MtGlb1-2.4*). Lengths of exons (without UTRs) and introns are given in base pairs (bp). Coding sequences (CDS in bp) and predicted protein sizes in number of amino acids (aa) are indicated on the right. Data were retrieved from the National Center for Biotechnology Information (NCBI; https://blast.ncbi.nlm.nih.gov/Blast.cgi) and the Phytozome portal, release v.12 (Mt4.0v1; https://phytozome.jgi.doe.gov). **(B)** Predicted heme domains for the four proteins according to the NCBI database. The scheme was drawn using Geneious v2019.0 created by Biomatters (https://www.geneious.com). **(C)** RNA-sequencing (RNA-seq) expression analysis of the four splice forms during nodule development and after NO_3_^−^ application. Nod0 are roots before inoculation; Nod4 are root bumps at 4 days post-inoculation (dpi); Nod10, Nod14, and Nod28 are nodules at 10, 14, and 28 dpi; and Nod14 + 12 h NO_3_^−^ and Nod14 + 48 h NO_3_^−^ are nodules at 14 dpi that were treated for 12 or 48 h with 10 mM KNO_3_. Data are means of three biological replicates with standard errors. **(D)** RNA-seq expression analysis of the four splice forms in other plant organs. For **(C,D)**, data are given as fragments per kb of transcript per million reads mapped (FPKM) and represent the means of three biological replicates with standard errors (raw data are indicated in Supplementary Table S1). For each alternative splice form, asterisks denote significant differences (Student’s *t*-test *p* < 0.05) with respect to the plant organ used as control: “Nod0” for panel **(C)** and “Leaf” for panel **(D)**.

**Figure 2:**
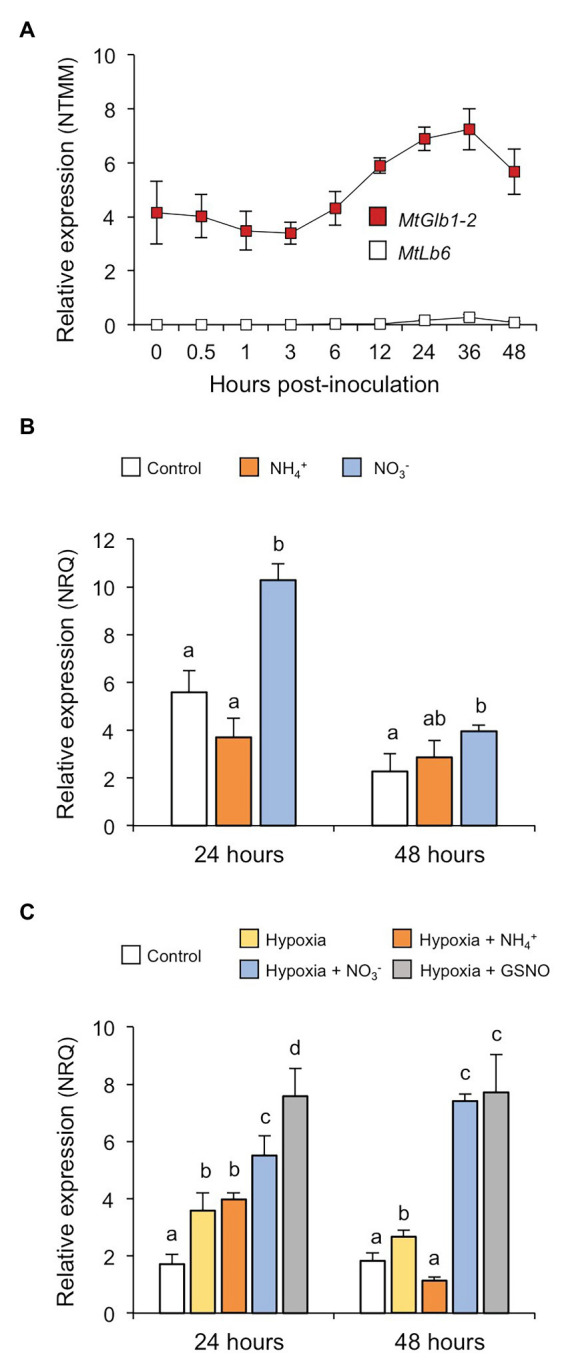
*MtGlb1-2* expression is induced by hypoxia and NO. **(A)** Expression profile of *MtGlb1-2* in roots after inoculation with *Sinorhizobium medicae* ABS7 (Larrainzar et al., 2015), now remapped to the latest *Medicago truncatula* genome version (Mt5.0). For comparison, the expression profile of a typical Lb gene (*MtLb6*) was included. Values are means with standard errors of four biological replicates for each time point. **(B)** Effect of NH_4_^+^ and NO_3_^−^ on *MtGlb1-2* expression in roots. Plants were grown for 11 days on Fahräeus plates and then transferred to fresh plates unsupplemented (controls) or supplemented with 2 mM NH_4_Cl or 2 mM KNO_3_. **(C)** Effect of hypoxia in the absence and presence of NH_4_^+^, NO_3_^−^, or *S*-nitrosoglutathione (GSNO) on *MtGlb1-2* expression in roots. Plants were grown for 11 days on Fahräeus plates and roots were submerged for 24 and 48 h in 5 mM phosphate buffer (pH 7.0) alone (hypoxia) or supplemented with 2 mM NH_4_Cl, 2 mM KNO_3_, or 2 mM GSNO. Controls were run in parallel by growing plants for 13 days on plates. Transcript levels were determined by qRT-PCR and expressed as normalized relative quantities (NRQs). Data are means of 3–5 biological replicates with standard errors. Statistical analyses were performed using log_2_(NRQ)-transformed data (Rubio et al., 2019) and comprised one-factor analysis of variance and one-tailed Student’s *t-*tests for mean comparisons. Means denoted by the same letter do not differ at *p* < 0.05.

For quantitative reverse transcription-polymerase chain reaction (qRT-PCR) analyses, total RNA was extracted from roots using the RNAqueous isolation kit (Invitrogen), treated with DNase I (Roche, Penzberg, Germany), and cDNA was synthesized with MMLV-RT (Promega, Madison, United States). qRT-PCR was performed using *MtGlb1-2* specific primers (5'–CTCAACTTCAATAAGTTTGTTTGGG–3' and 5'–ACTTATGCTAGAATCACATCTACCT–3'; efficiency 95%) with a 7500 Real-Time PCR System (Applied Biosystems, Waltham, United States), as previously described (Rubio et al., 2019). Normalized relative quantities were obtained using the geometric mean of the reference genes *Mt26SProt* (Medtr5g022440; 5'–TGGCAGGAAAGGGTGTTC–3' and 5'–GCCACCTGAATACCAGCAG–3'; efficiency 96%) and *MtPTB2* (Medtr3g090960; 5'–CGCCTTGTCAGCATTGATGTC–3' and 5'–TGAACCAGTGCCTGGAATCCT–3'; efficiency 95%). Plant treatments and statistical analyses are indicated for each type of experiments in the legends of Figures 1, 2.

### cDNA and Promoter Cloning, Hairy Root Transformation, and Histochemical Localization

The coding regions of *MtGlb1-2.1* and *MtGlb1-2.4* were amplified from a nodule cDNA library using specific forward primers for *MtGlb1-2.1* (5'–CACCATGGAAGAGAACAAGAAAACTGTG–3') and *MtGlb1-2.4* (5'–CACCATGTGGTCTCTAGCTATGAA–3'). The reverse primer was the same for both splice forms (5'–TTAAGAGGAGGGTTTGGATTTG–3'). The PCR products were cloned into the pENTR/D-TOPO vector (Invitrogen) and the correct sequences and orientations were verified by sequencing. To clone the promoter, DNA fragments containing 0.89 kb upstream of the predicted coding region of *MtGlb1-2.1* were cloned into the binary vector pBGWFS7 (Karimi et al., 2002) using the Gateway system to make transcriptional fusions with the *uidA* gene. This relatively small promoter length was chosen to avoid overlapping with the coding region of Medtr4g068880, which is located directly upstream on chromosome 4. The empty vector pBGWFS7 was used as a negative control for the transformations. Plasmids were electroporated into *Agrobacterium rhizogenes* ARqua1, and transformed hairy roots were generated in *M. truncatula* as described by Boisson-Dernier et al. (2001).

The histochemical localization of the *MtGlb1-2* promoter activity was performed using fusions with the *gus* reporter gene. Four-week-old transformed plantlets were transferred to pots containing a 1:1 perlite:vermiculite mixture and inoculated with *S. meliloti* 1021 constitutively expressing *Ds*Red to co-localize gene expression and infection. Samples were harvested at 2 and 4 weeks post-inoculation. Prior to staining for β-glucuronidase (GUS) activity, roots were prefixed under vacuum in 0.3% (w/v) paraformaldehyde in 100 mM sodium phosphate buffer (pH 7) for 30 min. Roots were washed in buffer and immersed in GUS staining solution: 1 mg ml^−1^ X-Gluc, 2.5 mM potassium ferricyanide, 2.5 mM potassium ferrocyanide, 10 mM EDTA, 0.1% (v/v) Triton X-100, and 100 mM sodium phosphate (pH 7.0). They were then vacuum-infiltrated for 20 min and incubated overnight at 37°C in the dark. Roots and nodules were washed several times in 70% ethanol and then in phosphate buffer. They were cut and mounted on glass slides in 50% glycerol (prepared in buffer) for microscopic observation. Nodules were embedded in 5% (w/v) agarose and 60-μm sections made with a vibratome. Root and nodule preparations were visualized with a Leica M165 FC stereomicroscope using transmitted light or with a DSR filter (excitation 545/30 nm and emission 620/60 nm) to detect the red fluorescence emitted by the bacteroids.

### Production and Purification of Recombinant Globins

The MtGlb1-2.1 and MtGlb1-2.4 proteins and their mutant derivatives were cloned into pET30(a) + or pET11a expression vectors (Novagene) with an N-terminal His-tag (HT) or Strep-tag (ST), respectively. Both sets of proteins were expressed in *E. coli* C41(DE3) cells (Lucigen). Cells were precultured overnight at 37°C with mild agitation (200 rpm) in 100 ml of LB medium with 100 μg ml^−1^ ampicillin or 100 μg ml^−1^ kanamycin, depending on the vector. To 1 l of TB medium, 10 ml of the preculture was added and cells were incubated for 24 h in the case of WT globins and until an OD at 600 nm of 0.5–0.8 was reached in the case of mutant globins. Then, 0.25 mM isopropyl β-D-1-thiogalacto-pyranoside was added to the medium and cells were grown at 28°C for 16 h. Transformed cells were washed in phosphate buffer and stored at −80°C for less than 3 weeks. For purification of ST proteins, cells were resuspended in 20 mM Tris (pH 8.0) with 150 mM NaCl, sonicated (3 × 2 min), and cleared by centrifugation. The supernatant was loaded on a StrepTactin Sepharose High Performance column (GE Healthcare) previously equilibrated with the same buffer. After loading the protein, the column was washed with at least five volumes of buffer and the recombinant proteins were eluted with 2.5 mM desthiobiotin in buffer. For purification of HT proteins, supernatants were loaded on a HiTrap chelating HP Ni-affinity column (GE Healthcare). The column was washed with five volumes of the same medium and the recombinant proteins were eluted with 50 mM potassium phosphate (pH 7.5) containing 150 mM NaCl and 250 mM imidazole. The proteins were dialyzed, concentrated, desalted, oxidized with ferricyanide, and desalted again. Purified proteins were quantified in the ferric form using an extinction coefficient of 150 mM^−1^ cm^−1^ for the Soret band.

### UV-Vis and Resonance Raman Spectroscopies

The UV-Vis spectra of the ferric and deoxyferrous forms of MtGlb1-2.1 and MtGlb1-2.4, as well as the spectra of their complexes, were obtained as follows. The cyano-ferric globin complex was produced by adding a few crystals of KCN to the ferric globin in the cuvette; deoxyferrous globin by adding a trace of dithionite to ferric globin in the cuvette; and nitrosyl-globin by adding a few crystals of dithionite and NaNO_2_ (Calvo-Begueria et al., 2018). Oxyferrous globin was obtained by adding a trace of dithionite and passing the protein through a NAP-5 mini-column equlibrated with the same buffer. Spectra were recorded immediately after production of the globin complexes using a 0.1 cm cuvette with a Lambda 25 spectrophotometer (Perkin-Elmer). Globin buffers and concentrations are indicated in the legends of Supplementary Figures S2, S3.

Resonance Raman (RR) measurements were performed with 25–30 μM globin in 20 mM Tris (pH 8.0) containing 150 mM NaCl. Deoxyferrous globins were prepared at pH 8.0 by addition of 2–3 μl of a freshly prepared sodium dithionite solution (20 mg ml^−1^) to the ferric globin solution (40 μl) previously flushed with N_2_. UV-Vis spectra of ferric and deoxyferrous globins were measured both prior and after the measurements to ensure that no degradation occurred under our experimental conditions. Absorption spectra were recorded in a 5-mm NMR tube (300 nm min^−1^ scan rate) or a 1-mm cuvette (600 nm min^−1^ scan rate) at 25°C. For the second derivative spectra (*D*^2^), the Savitzky-Golay method was applied using 15 data points (LabCalc, Galactic Industries, Salem). No changes in the wavelength or in the bandwidth were observed when the number of points was increased or decreased. RR spectra were obtained at 25°C using a 5-mm NMR tube by excitation at the 406.7 and 413.1 nm lines of a Kr^+^ laser (Coherent Innova 300C, Santa Clara, CA, United States), and at the 441.6 nm line of a He-Cd laser (Kimmon IK4121R-G, Kimmon Koha Co., Tokyo, Japan). Back-scattered light from a slowly rotating NMR tube was collected and focused into a triple spectrometer (consisting of two Acton Research SpectraPro 2300i instruments and a SpectraPro 2500i instrument in the final stage with gratings of 3,600 and 1,800 grooves/mm) working in the subtractive mode, equipped with a liquid nitrogen-cooled CCD detector. A spectral resolution of 1.2 cm^−1^ with a spectral dispersion of 0.40 cm^−1^/pixel, and of 4 cm^−1^ with a spectral dispersion 1.2 cm^−1^/pixel were calculated theoretically on the basis of the optical properties of the spectrometer for the 3,600 and 1,800 gratings, respectively. The RR spectra were calibrated with indene and carbon tetrachloride as standards to an accuracy of 1 cm^−1^ for intense isolated bands. All RR measurements were repeated several times under the same conditions to ensure reproducibility. To improve the signal-to-noise ratio, a number of spectra were accumulated and summed only if no spectral differences were noted. All spectra were baseline-corrected.

### Electron Paramagnetic Resonance Spectroscopy

For EPR analyses, samples containing 100–500 μM ferric globin and 20% glycerol were transferred to EPR quartz tubes (3-mm diameter), frozen, and stored in liquid nitrogen until use. EPR measurements were performed on a Bruker Elexsys spectrometer operating at X-Band (microwave frequency ~9.6 GHz). The experiments were performed at low temperatures by means of a He gas-flow cryostat and a temperature controller, both from Oxford Instruments. All the continuous wave (CW)-EPR spectra were taken both at 9 and 15 K in order to compare the contributions from high spin and low spin heme species. Microwave power was adjusted to ensure that there was no saturation, typically 2 mW. Modulation frequency and amplitude of the magnetic field were 100 kHz and 0.4 mT, respectively. Pulse EPR experiments (three pulse echo-induced EPR) were taken at 15 K. The μw pulse sequence was (π/2-*τ*-π/2-*t*-π/2), with *τ* = 240 ns and *t* = 400 ns. Echo was detected at a fixed position during a field sweep. Suitable phase cycling was applied to remove unwanted echoes.

### Nanosecond Laser Flash Photolysis

Changes in absorbance following laser dissociation of O_2_ were measured with an Applied Photophysics LKS80 laser flash photolysis (LFP) spectrometer coupled with a Brilliant B Nd-Y laser (second harmonic, 532 nm) from Quantel. Averages of 6–8 flashes were recorded and observed rate constants calculated by using the ProKinetist software.

### Nitric Oxide Binding Kinetics

For all reactions involving NO or NO_2_^−^, globins were prepared in 50 mM phosphate buffer (pH 7.0) and 150 mM NaCl. Degassed deoxyferrous globin was transferred anaerobically to a 10 ml syringe and mixed (1:1) with NO, prepared from the addition of the NO donor Proli-NONOate (Cayman) to degassed buffer. The concentration of Proli-NONOate was checked by UV spectroscopy using an extinction coefficient at 252 nm of 8,400 M^−1^ cm^−1^ (Maragos et al., 1991). The final concentrations of deoxyferrous globin and NO were 2.5 and 10–160 μM, respectively. The conversion of deoxyferrous to nitrosyl globin was followed at 20°C for up to 2 s with an Applied Photophysics SX20 stopped-flow instrument fitted with a diode array spectrophotometer.

### Nitric Oxide Dioxygenase Activity

To detect NOD activity, oxyferrous globin was produced by passing the deoxyferrous form through a Sephadex G-25 mini-column (NAP-5). The oxyferrous globins (50 μM) in 50 mM potassium phosphate buffer (pH 7.0) were immediately mixed with 0.1 or 1 mM diethylamine-NONOate (DEA-NO; Merck). The UV-Vis spectra were then taken after 5 s and 5 min with a Lambda 25 spectrophotometer (Perkin-Elmer). For NOD measurements, phosphate buffer was degassed using a glass tonometer connected to a supply of Ar gas and a vacuum pump, and it was transferred anaerobically to a 10 ml glass Hamilton syringe. Solutions containing NO were prepared by injecting into the degassed buffer using a Hamilton syringe. Oxyferrous globin was rapidly mixed (1:1) with NO at final concentrations of 2.5 and 5–160 μM, respectively, using the stopped-flow instrument mentioned earlier, pre-cooled to 10°C. The conversion of oxyferrous to ferric globin was completed within ms.

### Nitrite Reductase Activity

Degassed deoxyferrous globin was treated with NaNO_2_ dissolved in buffer in the presence of an excess of sodium dithionite. The final concentrations of deoxyferrous globin and NaNO_2_ were 2.5 μM and 0.05–1 mM, respectively. Reactions were performed at 25°C in 50 mM sodium phosphate buffer (pH 7.0) containing 150 mM NaCl. Optical spectra were recorded using an Agilent 8453 diode array spectrophotometer. Rate constants were determined from the time courses (0–200 s) by following the conversion of deoxyferrous to ferric globin in the Soret band and then by fitting the data to exponential functions using the least square method using the Microsoft Excel solver program.

## Results and Discussion

### A Plant Gene Encoding One-Heme and Two-Heme Globins Is Induced by Hypoxia and Nitric Oxide

The Medtr4g068870 gene of *M. truncatula* was designated here as *MtGlb1-2* because the encoded proteins have homology with class 1 Glbs of other legumes or model plants (Supplementary Figure S1; Berger et al., 2020b; Larrainzar et al., 2020). A BLAST search in the National Center for Biotechnology Information (NCBI) indicated certain similarity between the MtGlb1-2 proteins and some Glbs of the subterranean clover (*Trifolium subterraneum*, access code GAU15715; 335 amino acids and 85% identity) and the tropical tree *Cinnamomum micranthum* (access code RWR91940; 326 amino acids and 61% identity). However, in sharp contrast to any other hemoglobin gene retrieved from the databases, the precursor RNA of *MtGlb1-2* potentially gives rise to four alternative splice forms, which are named here *MtGlb1-2.1* to *MtGlb1-2.4* (Figure 1A). Gene modeling predicts that *MtGlb1-2.1* and *MtGlb1-2.2* are encoded by eight exons and differ only by 21 bp in exon 5, whereas *MtGlb1-2.3* is encoded by exons 2–8 and *MtGlb1-2.4* by part of exon 4 and exons 5–8. As for the proteins, MtGlb1-2.1 and MtGlb1-2.2 are predicted to have two heme domains, MtGlb1-2.4 only one, and MtGlb1-2.3 one or two (Figure 1B). To demonstrate the presence of the four splice variants, we performed RNA sequencing (RNA-seq) of nodules (Figure 1C) and other plant organs (Figure 1D). Results reveal that the four predicted splice variants are predominantly expressed in nodules and roots, with reduced expression levels in leaves. Very low expression was detected in pods (Figure 1D) and negligible expression in stems, petioles, buds, and flowers (data not shown). *MtGlb1-2.2* was the most abundantly expressed form in nodules and roots, but significant expression of *MtGlb1-2.1* in roots and of *MtGlb1-2.3* in nodules was also observed (Figures 1C,D). Expression of *MtGlb1-2.4* was very low or undetectable in most plant organs. Notably, the expression of *MtGlb1-2.2* was induced in nodules following supply with 10 mM KNO_3_ for 12 h (Figure 1C).

The expression of *MtGlb1-2* is already detectable during the first 2 days of infection, whereas a typical *Lb* gene, such as *MtLb6* (Medtr5g066070; Larrainzar et al., 2015), is not expressed in uninoculated roots or within the first hours of inoculation (Figure 2A). To gain insight into the function of *MtGlb1-2*, we quantified its expression in roots exposed to different nitrogen sources and/or to different conditions of O_2_ availability (Figures 2B,C). Two experiments were carried out including nitrate (NO_3_^−^) as a precursor of NO (Gupta and Igamberdiev, 2016) and ammonium (NH_4_^+^) as a negative control because it is not oxidized to NO in plant tissues. In the first experiment, plants were grown on plates for 11 days, transferred to new plates with no nitrogen or with NH_4_^+^ or NO_3_^−^, and harvested after 24 or 48 h (Figure 2B). At both time points, NH_4_^+^ had no effect but NO_3_^−^ induced *MtGlb1-2*. In a second experiment, we examined the effect of hypoxia by waterlogging. Plants were grown for 11 days in plates and then roots were submerged in buffer containing or not NH_4_^+^, NO_3_^−^, or *S*-nitrosoglutathione (GSNO) for 24 or 48 h (Figure 2C). In this experiment roots were protected from light as GSNO is a photolabile NO-releasing compound (Smagghe et al., 2008). *MtGlb1-2* was induced after 24 h of hypoxia and this induction was unaffected by the presence of NH_4_^+^ but further enhanced by NO_3_^−^ or GSNO. After 48 h, the expression level of *MtGlb1-2* with NH_4_^+^ was similar to that of normoxic roots but the gene remained consistently upregulated in the presence of NO_3_^−^ or GSNO. We thus conclude that *MtGlb1-2* is responsive to a source of NO such as NO_3_^−^ or GSNO, especially under low O_2_ conditions.

### The *MtGlb1-2* Gene Is Predominantly Expressed in the Meristems and Vascular Bundles of Roots and Nodules

**Figure 3:**
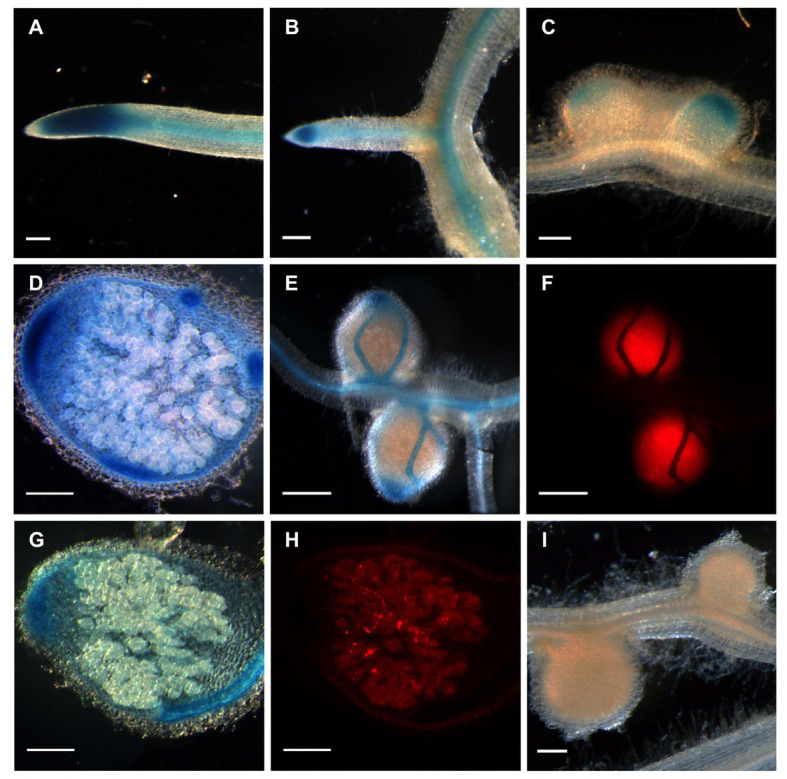
Localization of *MtGlb1-2* promoter activity gene in *Medicago truncatula* roots and nodules. The promoter region was cloned into the binary vector pBGWFS7 (Karimi et al., 2002) to make promoter: GUS fusions. Roots expressing the construct were produced by hairy root transformation and plants were inoculated with *Sinorhizobium meliloti* expressing *Ds*Red. **(A,B)** GUS activity is observed in the apex and vascular bundles of the primary root and a lateral root. **(C)** In young nodules, about 2 weeks post-inoculation (wpi), expression is readily visible also in the apex, encompassing zones I and II. **(D,E,G)**. At later stages (4 wpi), expression is readily visible also in the vascular bundles. In **(F)** and **(H)**, GUS activity does not appear to co-localize with infected cells. **(I)** Nodulated roots (2 wpi) corresponding to plants transformed with the empty vector pBGWFS7 as a negative control. Images **(A–E,G,I)** were taken with transmitted light. Images **(F)** and **(H)** correspond to nodules shown in **(E)** and **(G)**, respectively, and were taken with a DSR filter (Leica). Scale bars, 200 μm.

More functional information of *MtGlb1-2* was obtained by localizing the promoter activity in roots and nodules. To this end, we fused the promoter with the *uidA* gene, transformed hairy roots and inoculated them with *S. meliloti* 1021 constitutively expressing *Ds*Red. In primary and lateral roots, intense GUS activity staining was found in the meristem, elongation zone, and vascular bundles (Figures 3A,B). In nodules, GUS staining was observed predominantly in the meristem, distal region of infection zone, and vascular bundles (Figures 3C–E,G), but did not colocalize with infected cells, which were easily distinguishable by the red-tagged bacteroids (Figures 3F,H). Control nodules formed on roots transformed with the corresponding empty vector were completely devoid of GUS staining (Figure 3I). Because the meristems (Mira et al., 2016) and vascular bundles (Gaupels et al., 2008) are plant tissues with high respiratory activity and strict NO homeostasis, the localization of *MtGlb1-2* activity also supports a role related to O_2_ and NO for the protein products.

Our data of transcript quantification, including RNA-seq analyses of the alternative splice forms, as well as the localization of promoter activity in roots and nodules, indicate that *MtGlb1-2* is fully functional and is induced by hypoxia and NO. This led us to explore the reactivity of the proteins toward O_2_, NO, and NO_2_^−^ using biochemical and biophysical methods. Those studies were focused on MtGlb1-2.1 and MtGlb1-2.4 as representative proteins with two hemes and one heme, respectively, and their results are reported in the next sections.

### Heme Environment in Wild-Type and Mutated Globins

To study the heme configurations and reactions with physiological ligands, we cloned *MtGlb1-2.1* and *MtGb1-2.4* and produced the recombinant proteins. Cloning of the splice forms from the nodule cDNA library confirmed the RNA-seq data, demonstrating their presence *in vivo*. We produced both the WT versions and mutant variants in which the distal His (His_d_) was replaced by Leu. The numbers indicate the His_d_ removed, starting from the initial methionines (Supplementary Figure S1): 74, 238, and 74/238 for the MtGlb1-2.1 mutants and 109 for the MtGlb1-2.4 mutant. Two versions of the proteins, with HT and ST at the N-terminus, were purified by affinity chromatography. The mutated proteins were stable only when 150 mM NaCl was included in the medium and all the proteins were therefore purified in saline buffer to facilitate comparisons. Although the HT and ST versions of each protein displayed identical UV-Vis (Supplementary Figures S2, S3) and RR (Supplementary Figure S4) spectra, we decided to use the HT proteins in all our experiments because several ST proteins exhibited significant endogenous fluorescence that interfered with RR measurements.

**Figure 4:**
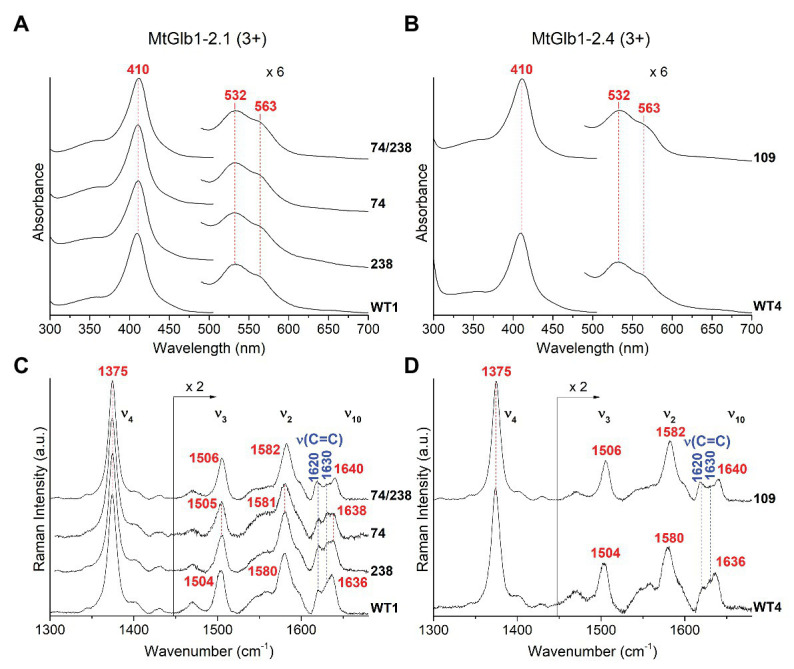
Spectroscopic characterization of the ferric form (3+) of MtGlb1-2.1 and MtGlb1-2.4. **(A,B)** UV-Vis and **(C,D)** RR spectra in the high frequency region of ferric WT1, WT4, and their mutants. The wavelength of the UV-Vis bands and the wavenumbers of the RR core size marker bands of the 6cLS species and the RR vinyl *v*(C=C) stretching modes are reported in red and blue, respectively. The spectra have been shifted along the *y*-axis. The 490–700 nm region of the UV-Vis spectra and the 1,450–1,700 cm^−1^ region of the RR spectra are expanded 6‐ and 2-fold, respectively, to facilitate visualization. The intensity of the RR spectra is normalized to the *v*_4_ band. RR experimental conditions: laser excitation 406.7 nm; laser power at the sample, 5 mW; and number of spectra used to calculate the average spectrum x integration time in minutes: **(C)** WT1 (4 × 40), 74 (4 × 40), 74/238 (4 × 40), and 238 (7 × 70). **(D)** WT4 (6 × 60) and 109 (4 × 40).

The proteins were characterized by UV-Vis, RR, and EPR spectroscopies. The ferric Glbs show UV-Vis spectra that are characteristic of a hexacoordinate low-spin (6cLS) heme, with Soret at ~410 nm and Q bands at 532 nm with a shoulder at 563 nm (Figures 4A,B). The spectra of MtGlb1-2.1 and MtGlb1-2.4 (WT and mutants) obtained with excitation in the Soret band at 406.7 nm (or at 413.1 nm) show core size marker band frequencies characteristic of a 6cLS heme (Spiro and Li, 1988; ν_3_, ν_2_, and ν_10_ in Figures 4C,D). Interestingly, the spectra of the 74/238 and 109 mutants show slightly up-shifted core size frequencies. The bandwidths in the WT spectra are slightly larger than those in the mutants, suggesting that two 6cLS hemes, with similar but not identical frequencies, are overlapped in the WT sample. The double mutant seems to be a pure single 6cLS. The vinyl stretching modes [ν(C=C), *in blue*] at 1,620 and 1,630 cm^−1^ (Figures 4C,D) are identical for all the spectra. The frequencies are sensitive to their orientations with respect to the porphyrin plane (Marzocchi and Smulevich, 2003), indicating *trans* and *cis* configurations, respectively (Milazzo et al., 2020).

**Figure 5:**
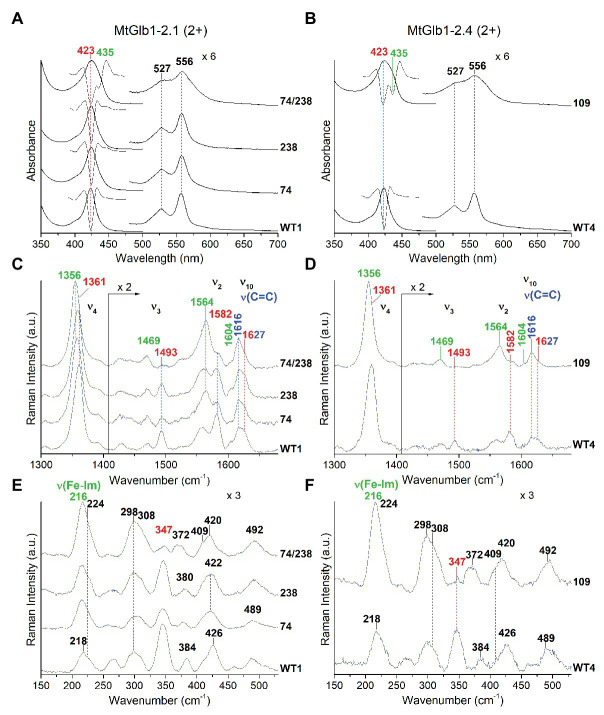
Spectroscopic characterization of the deoxyferrous form (2+) of MtGlb1-2.1 and MtGlb1-2.4. **(A,B)** UV-Vis and second derivative (*D*^2^) spectra in the Soret region together with RR spectra in the **(C,D)** high and **(E,F)** low frequency regions of deoxyferrous WT1, WT4, and their mutants. RR spectra were obtained with excitation at 441.6 nm. The wavelength of the Soret band and the wavenumbers of the RR marker bands of the 6cLS and 5cHS species and the RR vinyl *v*(C=C) stretching modes are reported in red, green, and blue, respectively. The spectra have been shifted along the *y*-axis. The 475–700 nm region of the UV-Vis spectra and the 1,430–1,700 cm^−1^ region of the RR spectra are expanded 6‐ and 2-fold, respectively, to facilitate visualization. The intensity of the RR spectra is normalized to the *v*_4_ band. RR experimental conditions: laser power at the sample 10 mW; and number of spectra used to calculate the average spectrum x integration time in minutes: **(C)** WT1 (3 × 9), 238 (6 × 18), 74 (6 × 30), and 74/238 (4 × 10). **(D)** WT4 (3 × 9) and 109 (6 × 18). **(E)** WT1 (4 × 20), 238 (3 × 18), 74 (4 × 16), and 74/238 (4 × 16). **(F)** WT4 (2 × 16) and 109 (3 × 15).

In the deoxyferrous form, the spectra of WT1 and WT4 are identical and characteristic of 6cLS heme, with Soret at 422 nm and Q bands at ~527 and ~556 nm (Figures 5A,B). However, in the 74/238 and 109 mutants, the spectra show the presence of pentacoordinate high-spin (5cHS) heme. This is evidenced by the broadening of the Soret and Q bands. The second derivative spectra (*D*^2^) in the Soret region confirm the presence of two bands at 423 nm (low spin) and 435 nm (high spin). The deoxyferrous proteins were therefore examined using laser excitations at 413.1 nm, in resonance with a 6cLS heme (Soret at ~422 nm), and at 441.6 nm, in resonance with a 5cHS heme (Soret at *~*435 nm), in order to selectively intensify the 6cLS and 5cHS core size marker bands, respectively. The WT1 and WT4 proteins show identical high frequency RR spectra with both excitations (Figures 5C,D and Supplementary Figures S5, S6), characteristic of a 6cLS heme (*core size markers in red*). In contrast, in both 74/238 and 109 mutants, strong bands due to a 5cHS heme (mainly intensified with excitation at 441.6 nm; *core size markers in green*) coexist with the 6cLS heme (mainly intensified with the 413.1 nm excitation), in agreement with the UV-Vis data. In the low frequency region of only 5cHS heme proteins obtained with excitation at 441.6 nm, an intense band due to the ν(Fe-Im) stretching mode is usually identified between 200 and 250 cm^−1^. Its frequency is an optimum probe of the proximal cavity structure as it is very sensitive to the protein matrix (Smulevich et al., 2010). Accordingly, in the low frequency region spectra of the 74/238 and 109 mutants obtained with excitation at 441.6 nm (Figures 5E,F and Supplementary Figure S7), the intense band at 216 cm^−1^ is assigned to the ν(Fe-Im) stretching mode. This frequency is similar to that observed for other plant hemoglobins (Ioanitescu et al., 2005).

Further confirmation that the ferric WT and mutant proteins have predominantly 6cLS hemes was obtained by EPR (Supplementary Figure S8). The spectra of both WT1 and WT4 show peaks in the 200-250 mT field region, which can be associated with a low spin center (Alonso et al., 2007). The principal values of the *g* tensor of the detected heme center are *g*_z_ = 3.08, *g*_y_ = 2.2, and *g*_x_ = 1.2. These values and the shape of the signals differ from the heme centers detected in other class 1 Glbs (Ioanitescu et al., 2005) and allow classifying it as a “highly anisotropic low spin” (HALS) center (Alonso et al., 2007), a less stable heme configuration caused by some hindrance of the protein structure to accommodate the His_d_ as the heme sixth ligand, which is consistent with the peculiarities of the His_d_ binding kinetics that are analyzed in the next sections.

### Extremely High Oxygen Affinities of Globins

**Figure 6:**
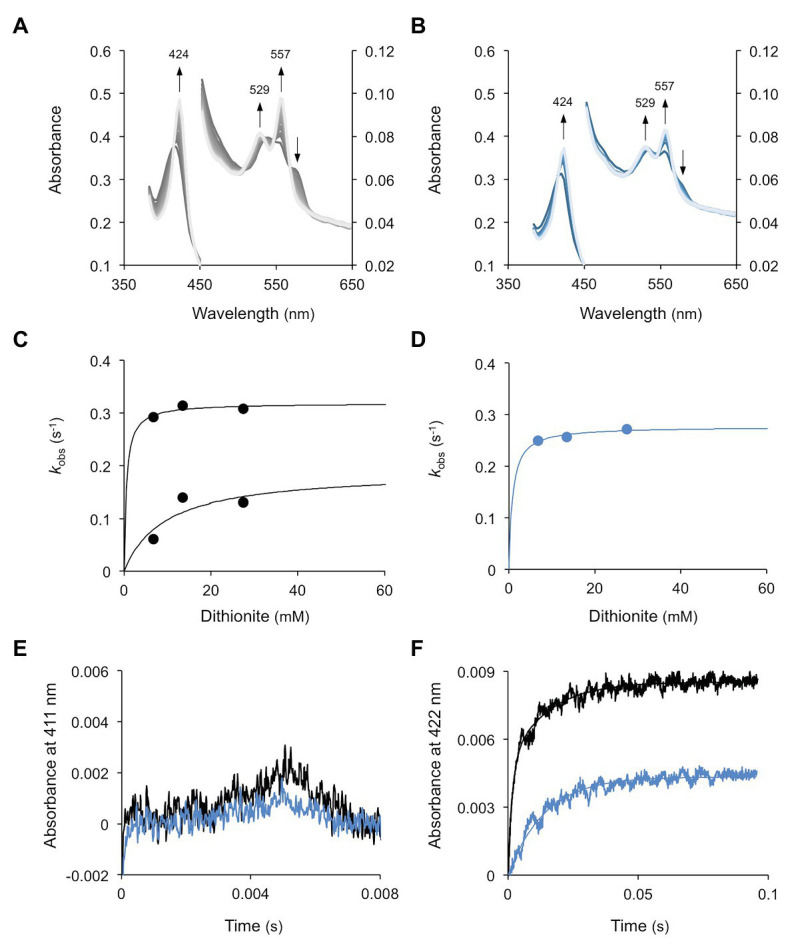
Dissociation and rebinding of O_2_ in WT1 and WT4. **(A)** Oxyferrous WT1 was mixed with dithionite and spectra were taken every 200 ms. Measurements were made in triplicate for each of the three concentrations of dithionite (6.75, 13.5, and 27.5 mM) and were fitted to a hyperbola function to obtain the maximum *k*_off(O2)_. **(B)** Similar experiments for WT4, but spectra were taken every 400 ms. **(C,D)** Oxygen dissociation kinetics, where two phases and a single phase were observed, respectively, for **(C)** WT1 and **(D)** WT4. Pseudo first order constants were plotted as a function of exogenous dithionite concentration. **(E)** LFP kinetics of the oxyferrous forms of WT1 (*black*) and WT4 (*blue*). **(F)** LFP kinetics of the carboxy forms of WT1 (black) and WT4 (blue) showing double and single exponential kinetics, respectively. The CO concentration was 500 μM. All experiments were performed at 25°C.

The O_2_ dissociation rate constants for WT1 and WT4 were determined by rapidly mixing each oxyferrous complex with a solution of sodium dithionite in a stopped flow spectrophotometer. Dithionite reacts rapidly with free O_2_ in solution but not with that bound to the heme Fe. The formation of the deoxy species of WT1 (Figure 6A) and WT4 (Figure 6B) therefore proceeds at the rate of O_2_ dissociation from the Fe. The pseudo first order rate constants, obtained by fitting the time courses to either a single or double exponential function, were essentially independent of dithionite concentration and reached a limiting value that represents the first order O_2_ dissociation rate constant. For WT1, the time courses were biphasic yielding two dissociation constants, which is consistent with the presence of two non-equivalent hemes (Figure 6C), whereas for WT4 the time courses were monophasic, as expected for a protein with a single heme (Figure 6D). The dissociation rate constants (*k*_off_) were calculated as 0.319 and 0.190 s^−1^ for WT1 and 0.277 s^−1^ for WT4.

Following LFP of oxyferrous WT1 and WT4 (Figure 6E), we did not observe recombination of O_2_ with the heme in the time frame of the experiment (>5 μs, the detector dead time). This was true both at high (~230 μM) and low (~20 μM) O_2_ tensions, which can be explained by the very low quantum yield for O_2_ photolysis together with the extremely rapid rate of recombination. To overcome the first of these two factors, the experiment was repeated in the presence of 500 μM CO. Under these conditions a fraction of the protein exists in the carboxy form. Upon photolysis the CO was dissociated and O_2_, free in solution, was able to bind to the heme. Again no fast phase for O_2_ recombination was observed although, in the millisecond-to-second time scale, slower processes were seen. These time courses had amplitudes with spectral distributions consistent with oxy to carboxy transitions and rate constants identical to the *k*_off_ values indicated earlier. These results show that O_2_ binds very rapidly following CO photolysis and is then displaced by CO in the dark. They also indicate that the O_2_ binding rate constants (*k*_on_) for both WT1 and WT4 were higher than those that can be observed with our laser photolysis/detector system. Hence, the estimated minimum *k*_on_ of WT1 and WT4 is >5 × 10^8^ M^−1^ s^−1^. Addition of dithionite to the experiment described above removed all O_2_ from the system, leaving the proteins in the carboxy forms. Upon photolysis, recombination of CO with each protein was observed, which was unaffected by competition with O_2_ (Figure 6F). The time courses were fitted to double (WT1) or single (WT4) exponential functions, reflecting the double and single hemes for WT1 and WT4, respectively. These yielded second order rate constants for CO binding of 11.7 × 10^5^ (fast) and 1.32 × 10^5^ M^−1^ s^−1^ (slow) for WT1 and 1.43 × 10^5^ M^−1^ s^−1^ for WT4. Finally, the minimum O_2_ affinity constants (*k*_on_/*k*_off_) can be estimated as 1.6 × 10^9^ and 2.6 × 10^9^ M^−1^ for WT1 and 1.8 × 10^9^ M^−1^ for WT4.

### Extreme Reactivity of Globins With Nitric Oxide and Nitrite

**Figure 7:**
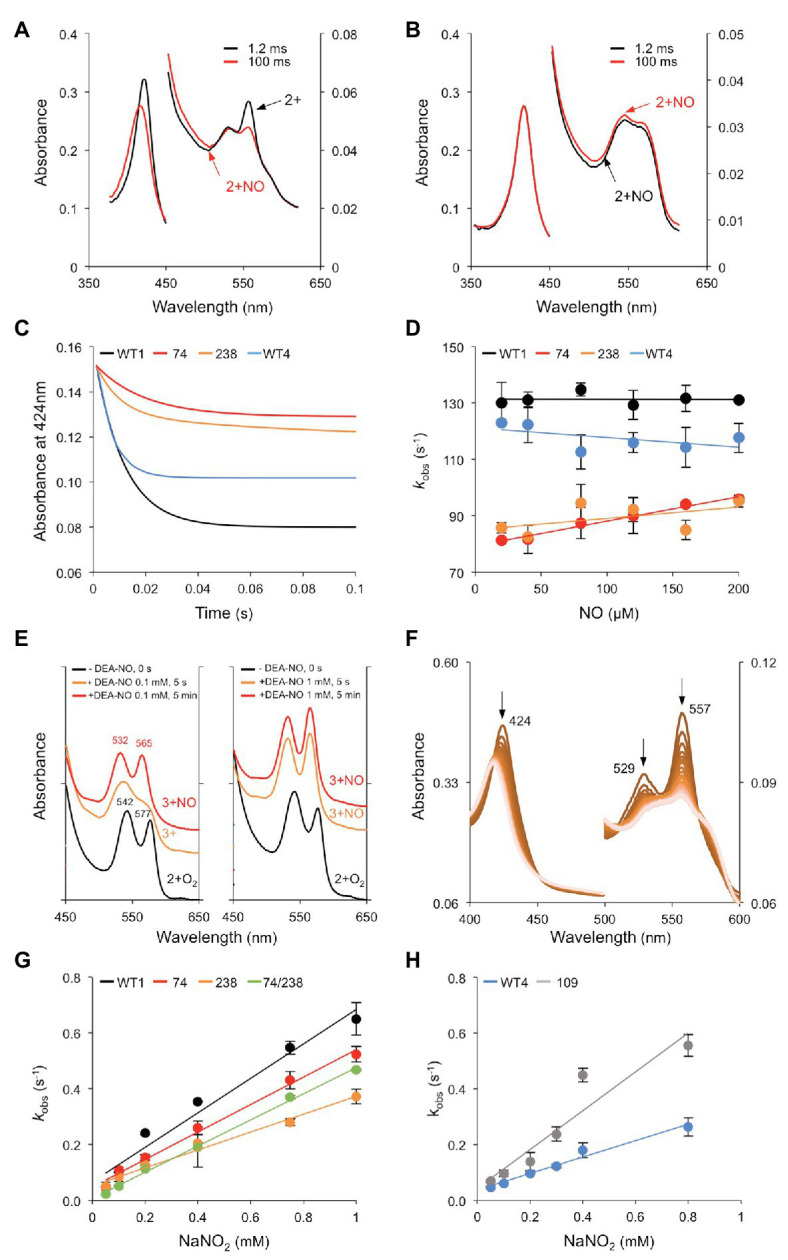
Reactivity of Glbs with NO and NO_2_^−^. **(A,B)** UV-Vis spectra of the reaction of 20 μM NO with 2.5 μM of the deoxyferrous forms of **(A)** WT1 and **(B)** 74/238 mutant. Spectra were taken at 1.2 and 100 ms after mixing. Note that, in the double mutant, the nitrosyl complex (2+NO) is already visible in the initial spectrum. **(C)** Time course of NO binding (20 μM) to the deoxyferrous proteins. Note that the amplitudes for the single mutants are lower than that of WT1 as only one of the hemes is observed with the other having already formed the nitrosyl complex (<1.2 ms). **(D)** Rate constants of NO binding were calculated from fitting the time course to a single exponential function. The panel shows the plots of observed pseudo first order rate constants (*k*_obs_) at pH 7.0 and 25°C vs. NO concentration. The second order bimolecular rate constant was obtained from the linear fit of the data. **(E)** NOD activity examined by UV-Vis spectroscopy. The oxyferrous form of WT1 (2+O_2_) was reacted with NO supplied as 0.1 or 1 mM diethylamine NONOate (DEA-NO), and the UV-Vis spectra were taken at the indicated times. The Soret bands and the peak wavelengths of the right plot (identical to those of the left plot) are omitted for clarity. Note the Q bands characteristic of the 3+NO complex. The WT4 protein showed identical behavior. **(F)** NiR activity of deoxyferrous WT1. The reaction was carried out with 50 μM NO_2_^−^ and 2.5 μM protein, generating 2+NO under anaerobic conditions. Spectra were taken every 1 s for 1 min, although only 46 spectra are shown for clarity. Note the decrease of the characteristic peaks at 424, 529, and 557 nm of the deoxyferrous globin. **(G,H)** Kinetics of the reactions between NO_2_^−^ and the ferrous forms of **(G)** WT1 and the 74, 238, and 74/238 mutants and **(H)** WT4 and the 109 mutant. The panels show the plots of observed pseudo first order rate constants (*k*_obs_) at pH 7.0 and 25°C vs. NO_2_^−^ concentration. The second order bimolecular rate constant was obtained from the linear fit of the data.

The reactivities of WT and mutated proteins with NO and NO_2_^−^ were examined because both molecules induce *MtGlb1-2* expression and play major roles in plants. Three types of reactions were measured: NO binding, NOD, and NiR (Figure 7). To study NO binding, the deoxyferrous proteins were mixed with NO and the reaction was followed by stopped flow spectroscopy. Formation of the nitrosyl complex was rapid for all the proteins but with some differences. For the WT1 protein, the deoxyferrous form was visible after the dead time (1.2 ms) of the instrument (Figure 7A), but by that time the deoxyferrous 74/238 had already been converted into the nitrosyl form (Figure 7B). The pseudo first order rate constants (*k*_obs_) were calculated from fitting the changes in absorbance at 424 nm to a single exponential plot (Figure 7C). The NO binding kinetics for WT1, 74, 238, and WT4 were found to be independent of NO concentration (Figure 7D) and the rate constants for NO binding (*k*_NO_) were estimated accordingly as 126, 88, 91, and 114 s^−1^, respectively. These rate constants are limited by the His_d_ dissociation rate constant and are in line with the His_d_ dissociation rate constants reported for other plant Glbs (Ioanitescu et al., 2005; Sainz et al., 2013; Leiva Eriksson et al., 2019). The 74/238 and 109 mutants displayed rapid NO binding (*k_NO_* > 250 s^−1^) because, in the absence of the His_d_, the rate of NO binding is not limited by the dissociation of this ligand from the iron.

Next, we examined the NOD activity of the proteins using UV-Vis spectroscopy. To this end, the oxyferrous WT1 and WT4 were exposed to 0.1 or 1 mM of the NO donor DEA-NO. The spectra were taken after 5 s (the dead time of the spectrophotometer) and after 5 min (Figure 7E). At a low NO concentration (0.1 mM DEA-NO), the oxyferrous was converted into ferric within 5 s and into ferric-NO after 5 min. At a high NO concentration (1 mM DEA-NO) the protein was already present as ferric-NO after 5 s. Because of the high NOD activity, we attempted to calculate the rates of WT1, WT4, and the mutant proteins by stopped flow. Considering the dead time of this instrument (1.2 ms) and that the time points are taken every ~1.2 ms, the time course of reactions with half times <3 ms will not be observed. As the first spectrum recorded after 1.2 ms was essentially that of a ferric globin, we conclude that *k*_NOD_ must be >250 s^−1^ even at NO concentrations as low as 5 μM. Controls omitting NO indicated that this very fast reaction was not due to autoxidation.

Finally, NiR reactions were assayed with NO_2_^−^ concentrations of 0.1–1 mM, at pH 7.0 and 25°C, in the presence of excess dithionite (Figure 7F). This avoids the formation of oxygenated globins and quickly reduces the resulting ferric form. The observed rate (*k*_obs_) for NiR activity was plotted against NaNO_2_ concentration and the second order rate (*k*_NiR_) was calculated from the slopes (Figures 7G,H). The *k*_NiR_ value of WT1 (726 M^−1^ s^−1^) was found to be approximately double that of WT4 (382 M^−1^ s^−1^), with the 74 mutant (534 M^−1^ s^−1^) and the 238 mutant (346 M^−1^ s^−1^), all showing bimolecular rate constants within a factor of ~2. Because only one rate constant was observed with WT1, it may be concluded that the two hemes show similar reactivity and kinetics toward NO_2_^−^. Under comparable assay conditions (pH 7, 0.1–2.5 mM NO_2_^−^, presence of dithionite), the NiR activities of the MtGlb1-2.1 and MtGlb1-2.4 proteins are between 5‐ and 10-fold higher than those reported for cyanobacterial SynHb (68 M^−1^ s^−1^) and for the class 1 Glbs of *Arabidopsis thaliana* (58 M^−1^ s^−1^) and rice (83 M^−1^ s^−1^; Sturms et al., 2011; Tiso et al., 2012). Comparison with animal hemoglobins is even more striking. The NiR activities of MtGlb1-2.1 and MtGlb1-2.4 are ~120-fold higher than for sperm whale myoglobin (2.9 M^−1^ s^−1^) and 2,800-fold higher than for human neuroglobin (0.25 M^−1^ s^−1^); in fact, they are only similar to the NiR activity of the His_d_ mutant of neuroglobin (956 M^−1^ s^−1^; Sturms et al., 2011; Tiso et al., 2011). It should be noted that the rate constant for the NiR reaction is highly pH dependent, with a logarithmic increase in rate as pH decreases because the protonated form of NO_2_^−^, nitrous acid (HNO_2_), is the active species (Doyle et al., 1981).

### Conclusions and Biological Relevance

In this work, we used a interdisciplinary approach to study a unique plant gene, *MtGlb1-2*, and their products belonging to the class 1 Glb family. The expression of *MtGlb1-2* is induced by low O_2_ and further enhanced in the presence of NO_3_^−^ or GSNO (Figure 2) and the gene promoter is active in root and nodule tissues (Figure 3). This indicates that the proteins perform both symbiotic and nonsymbiotic functions, probably linked to O_2_ and NO homeostasis. We attribute the increase in *MtGlb1-2* expression to NO, which may be released from GSNO or produced by the sequential reduction of NO_3_^−^ to NO_2_^−^ (in the cytosol) and of NO_2_^−^ to NO (in the cytosol and/or mitochondria). In fact, very recent studies with nodules of model legumes have shown that mutants in some NO_3_^−^ transporters (Valkov et al., 2020; Wang et al., 2020) or in NO_3_^−^ reductases (Berger et al., 2020a) display alterations in NO production and/or homeostasis. This clearly indicates that NO_3_^−^ is a major precursor of NO in nodules.

To gain insight into the Glb functions, we examined their reactions with heme ligands. Remarkably, we found extremely high reactivities of these proteins toward the physiological ligands O_2_, NO, and NO_2_^−^. The O_2_ association rates of MtGlb1-2.1 and MtGlb1-2.4 are extremely fast (>5 × 10^8^ M^−1^ s^−1^), although the dissociation rates are similarly slow to those of other class 1 Glbs (Smagghe et al., 2009). As a result, the O_2_ affinities of MtGlb1-2.1 and MtGlb1-2.4 are very high (estimated as <0.6 nM), meaning that the proteins remain oxygenated under normoxia or moderate hypoxia and probably become deoxygenated only under severe hypoxia. Both MtGlb1-2.1 and MtGlb1-2.4 have extremely fast NOD and NiR activities compared with the hemoglobins from vertebrates and other plants (Sturms et al., 2011; Tiso et al., 2011, 2012). However, the two reactions occur at opposite O_2_ levels and with opposite outcomes for NO metabolism. The NOD activity requires O_2_ and scavenges NO, whereas the NiR activity requires anaerobic (or nearly) conditions and generates NO. In the root and nodule cells, the NiR activity of Glbs is expected to be fast under hypoxic conditions because NO_2_^−^ accumulates (Gupta and Igamberdiev, 2016) and pH decreases (Roberts et al., 1984). Thus, an increase of NiR activity with lowering pH has been observed for human hemoglobin (Doyle et al., 1981) and neuroglobin (Tiso et al., 2011) and for *A. thaliana* class 1 and 2 Glbs (Tiso et al., 2012). On the other hand, the resulting ferric Glb from the NOD reaction could be recycled to ferrous Glb by NAD(P)H-reduced flavins because they are able to reduce ferric plant hemoglobins even under anoxic conditions (Becana and Klucas, 1990; Sainz et al., 2013). In conclusion, we propose an integrative model for the functioning of these novel Glbs by taking into account their transcriptional activation by low O_2_ and NO together with their extremely high reactivities toward diatomic gases and NO_2_^−^, which are reflected by their unusual NOD and NiR activities. In this model, we propose that the O_2_ tension within the tissue may poise Glbs to act as either NO scavengers or NO producers. Thus, Glbs offer a mechanism for very fast and fine tuning of NO concentration in the cytosol in response to sudden changes in O_2_ availability that may occur in the meristems during cell division and in the vascular bundles during metabolite transport in roots and nodules.

## Data Availability Statement

The original contributions presented in the study are included in the article/Supplementary Material, further inquiries can be directed to the corresponding author.

## Author Contributions

IV, GS, BR, RH, SA, MU, and MB designed experiments. IV, EL, LM, JIM, CP-R, MTW, MCR, SA, and MB performed experiments. GS, JIM, BR, and MB wrote the manuscript. All authors contributed to the article and approved the submitted version.

### Conflict of Interest

The authors declare that the research was conducted in the absence of any commercial or financial relationships that could be construed as a potential conflict of interest.
