# Corrigendum: A Plant Gene Encoding One-Heme and Two-Heme Hemoglobins With Extreme Reactivities Toward Diatomic Gases and Nitrite

**DOI:** 10.3389/fpls.2020.637797

**Published:** 2021-01-25

**Authors:** Irene Villar, Estíbaliz Larrainzar, Lisa Milazzo, Carmen Pérez-Rontomé, Maria C. Rubio, Giulietta Smulevich, Jesús I. Martínez, Michael T. Wilson, Brandon Reeder, Raul Huertas, Stefania Abbruzzetti, Michael Udvardi, Manuel Becana

**Affiliations:** ^1^Departamento de Nutrición Vegetal, Estación Experimental de Aula Dei, Consejo Superior de Investigaciones Científicas (CSIC), Zaragoza, Spain; ^2^Department of Sciences, Institute for Multidisciplinary Applied Biology, Campus Arrosadía, Universidad Pública de Navarra, Pamplona, Spain; ^3^Dipartimento di Chimica “Ugo Schiff”, Università di Firenze, Florence, Italy; ^4^Instituto de Ciencia de Materiales de Aragón, Universidad de Zaragoza-CSIC, Zaragoza, Spain; ^5^School of Life Sciences, Essex University, Wivenhoe Park, Colchester, United Kingdom; ^6^Noble Research Institute LLC, Ardmore, OK, United States; ^7^Dipartimento di Scienze Matematiche, Fisiche e Informatiche, Università di Parma, Parma, Italy

**Keywords:** *Medicago truncatula*, nitric oxide, symbiosis, phytoglobins, nodule, leghemoglobin, nitrate, hypoxia

In the original article, there were three errors. The concentrations of antibiotics should be expressed in micrograms per milliliter; the pH of the Tris buffer should be 8.0; and the concentration of deoxyferrous globin should be stated as 2.5 micromolar.

Two corrections have been made to: MATERIALS AND METHODS, Production and Purification of Recombinant Globins, paragraph 1, line 7 and line 17,

“…of LB medium with 100 μg ml^−1^ ampicillin or 100 μg ml^−1^ kanamycin,…”

“..20 mM Tris (pH 8.0) with 150 mM NaCl…”

One correction has been made to: MATERIALS AND METHODS, Nitrite Reductase Activity, paragraph 1, line 4,

“…were 2.5 μM and 0.05–1 mM, respectively.”

The authors apologize for this error and state that this does not change the scientific conclusions of the article in any way. The original article has been updated.

