# Supplementary material for: A Plant Gene Encoding One-Heme and Two-Heme Hemoglobins With Extreme Reactivities Toward Diatomic Gases and Nitrite
Source: Front Plant Sci. 2020 Nov 19;11:600336. doi: 10.3389/fpls.2020.600336 (PMC7710986; doi:10.3389/fpls.2020.600336)
Supplement: Supplementary file 1 [file Data_Sheet_1.pdf]

```
MtGlb1-2.3      -----------------------------------------------------MFSFLKDSNVPLEQNPKLKPHAMSVFLMTCESAVQLRKAGKVRVRESNLKKLGATHFKTGVQDEHFE      67
Atha1           ------------------------------------------------------------------------------------------------------------------------      0
Ljap1-1         ------------------------------------------------------------------------------------------------------------------------      0
Mtru1-1         ------------------------------------------------------------------------------------------------------------------------      0
Tpra1-1         ------------------------------------------------------------------------------------------------------------------------      0
Cari1-1         ------------------------------------------------------------------------------------------------------------------------      0
Lang1-1         ------------------------------------------------------------------------------------------------------------------------      0
MtGlb1-2.2      MEENKKTVDGSVDFTEEQEALVVKSWNAMKNNSCDLSLKFFTKILEIAPPAKQMFSFLKDSNVPLEQNPKLKPHAMSVFLMTCESAVQLRKAGKVRVRESNLKKLGATHFKTGVQDEHFE      120
MtGlb1-2.1      MEENKKTVDGSVDFTEEQEALVVKSWNAMKNNSCDLSLKFFTKILEIAPPAKQMFSFLKDSNVPLEQNPKLKPHAMSVFLMTCESAVQLRKAGKVRVRESNLKKLGATHFKTGVQDEHFE      120
MtGlb1-2.4      ------------------------------------------------------------------------------------------------------------------------      0
Gmax1-1         ------------------------------------------------------------------------------------------------------------------------      0
Pvul1-1         ------------------------------------------------------------------------------------------------------------------------      0
Vung1-1         ------------------------------------------------------------------------------------------------------------------------      0


MtGlb1-2.3      VTKQALLETIEEAIPEMWSLAMKNAWAEAHDQLANAIKVEMKEAHDQMDNANLIINMEENTGSCFTEEQEALVVKSWNAIKYNSGDLSLKFFKKILEIAPPAKQLFSFLKDSNVPLEHNP      187
Atha1           --------------------------------------------------------MESEGKIVFTEEQEALVVKSWSVMKKNSAELGLKLFIKIFEIAPTTKKMFSFLRDSPIPAEQNP      64
Ljap1-1         --------------------------------------------------------MSTLGSTCFTEEQEALVVKSWSVMKKNSAELGLKLFLKIFEIAPSAQKLFSFLRDSKVPLEENP      64
Mtru1-1         --------------------------------------------------------MGTLDTKGFTEEQEALVVKSWNAMKKNSAELGLKLFLKIFEIAPSAQKLFSFLKDSKVPLEKNT      64
Tpra1-1         --------------------------------------------------------MGTLETKGFTEEQEALVVKSWAVMKKNSAELGLKLFLKIFEIAPSAQKLFSFLKDSKVPLEQNT      64
Cari1-1         --------------------------------------------------------MDTLEKKGFTEEQEALVVKSWTAMKKNTAELSLKFFLKIFEIAPSAQKLFSFLKDSKVPLDQNP      64
Lang1-1         --------------------------------------------------------MSTLEVRGFTEEQEALVVKSWNAMKKNSGELALKFFLKIFEIAPSAQKMFSFLKDSQVPLEKNP      64
MtGlb1-2.2      VTKQALLETIEEAIPEMWSLAMKNAWAEAHDQLANAIKVEMKEAHDQMDNANLIINMEENTGSCFTEEQEALVVKSWNAIKYNSGDLSLKFFKKILEIAPPAKQLFSFLKDSNVPLEHNP      240
MtGlb1-2.1      VTKQALLETIEEAIPEMWSLAMKNAWAEAHDQLANAIKVEMKEAHDQMDNANLI-------SSCFTEEQEALVVKSWNAIKYNSGDLSLKFFKKILEIAPPAKQLFSFLKDSNVPLEHNP      233
MtGlb1-2.4      ----------------MWSLAMKNAWAEAHDQLANAIKVEMKEAHDQMDNANLIINMEENTGSCFTEEQEALVVKSWNAIKYNSGDLSLKFFKKILEIAPPAKQLFSFLKDSNVPLEHNP      104
Gmax1-1         --------------------------------------------------------MTTTLERGFSEEQEALVVKSWNVMKKNSGELGLKFFLKIFEIAPSAQKLFSFLRDSTVPLEQNP      64
Pvul1-1         ---------------------------------------------------------MNSSGKCFTEEQEALVLKSWSVMKKNSAELGLKFFLKIFEIAPSTQKLFSFLRDSTVPLEQNS      63
Vung1-1         ---------------------------------------------------------MNSMGKCFTEEQEALVVKSWNVMKKNSGELGLKFFLKIFEIAPSAQKLFSFLRDSTVPLEQNP      63
                                                                            *   * *   * ** * ** ****     **** **  *   * 

MtGlb1-2.3      KLKPHAMSVFLMTCESAVQLRKAGKVTVRESNLKKLGATHFKTGVKDEHFEVTKQALLETIKEALPEMWSPAMENAWGEAHDQLANAIKAEMKKTDHDHQTNVEDKSKPSS      298
Atha1           KLKPHAMSVFVMCCESAVQLRKTGKVTVRETTLKRLGASHSKYGVVDEHFEVAKYALLETIKEAVPEMWSPEMKVAWGQAYDHLVAAIKAEMNLSN---------------      160
Ljap1-1         KLKPHAMSVFVMTCESAAQLRKAGKVTVRESTLKKLGATHYKYGVVNEHFEVTKFALLDTIKEAVPEMWSPEMKNAWAQAYDQLVGAIKSEMKPSSS--------------      161
Mtru1-1         KLKPHAMSVFLMTCESAVQLRKSGKVTVRESSLKKLGANHFKYGVVDEHFEVTKFALLETIKEAVPEMWSPAMKNAWGEAYDQLVNAIKSEMKPSS---------------      160
Tpra1-1         KLKSHAMSVFLMTCESAVQLRKAGKVTVRESSLKKLGASHFKNGVVDEHFEVTKFALLETIKEAVPEMWSPEMKNAWGEAYDQLVNAIKSEMKPSS---------------      160
Cari1-1         KLKPHAMSVFLMTCESAIQLRKAGKVTVRESNLKKLGATHFKNGVVDEHFEVTKFALLETIKEAVPEMWSPAMKNAWGEAYDQLVDAIKTEMKASS---------------      160
Lang1-1         KLKTHALSVFVMTCESAVQLRKAGKVTVRESTLKKLGASHLKNGVVDEHFEVTKFALLETIKEAVPEIWSPAMKDAWGEAYDQLVGAIKSEMKPSS---------------      160
MtGlb1-2.2      KLKPHAMSVFLMTCESAVQLRKAGKVTVRESNLKKLGATHFKTGVKDEHFEVTKQALLETIKEALPEMWSPAMENAWGEAHDQLANAIKAEMKKTDHDHQTNVEDKSKPSS      351
MtGlb1-2.1      KLKPHAMSVFLMTCESAVQLRKAGKVTVRESNLKKLGATHFKTGVKDEHFEVTKQALLETIKEALPEMWSPAMENAWGEAHDQLANAIKAEMKKTDHDHQTNVEDKSKPSS      344
MtGlb1-2.4      KLKPHAMSVFLMTCESAVQLRKAGKVTVRESNLKKLGATHFKTGVKDEHFEVTKQALLETIKEALPEMWSPAMENAWGEAHDQLANAIKAEMKKTDHDHQTNVEDKSKPSS      215
Gmax1-1         KLKPHAVSVFVMTCDSAVQLRKAGKVTVRESNLKKLGATHFRTGVANEHFEVTKFALLETIKEAVPEMWSPAMKNAWGEAYDQLVDAIKSEMKPPSS--------------      161
Pvul1-1         KFKVHAISVFIMTCDSAVQLRKAGKVTVRESNLKKLGASHLRTGVVNEHFEVGKFALLETIKEAVPEMWSPEMKYAWGEAYDQLAEAIKSEMKPPSS--------------      160
Vung1-1         KLKPHATSVFVMTCDSAVQLRKAGKVTVRESTLKKLGATHLRTGVANEHFEVTKFALLETIKEAVPEMWSPEMKNAWGEAYDQLVDAIKSEMKPPSA--------------      160
                * * ** *** * * ** **** *******  ** *** *   **  ***** * ***  **** ** *** *  **  * * *  *** **
```

FIGURE S1 | Alignment of the four proteins encoded by the *MtGlb1-2* gene with typical class 1 Glbs of *Arabidopsis thaliana* and legumes. Highlighted in blue are the distal His residues. Alignment was performed with Clustal Omega from EMBL-EBI (https://www.ebi.ac.uk/Tools/msa/clustalo/). *Abbreviations and accession numbers:* Atha, *Arabidopsis thaliana* (At2g16060); Ljap, *Lotus japonicus* (Lj3g3v3338170); Mtru, *Medicago truncatula* (Medtr4g068860); Tpra, *Trifolium pratense* (PNX93138); Cari, *Cicer arietinum* (Ca_13651); Lang, *Lupinus angustifolius* (OIV99463); Gmax, *Glycine max* (Glyma.11G121800); Pvul, *Phaseolus vulgaris* (Phvul.011G048700); Vung, *Vigna unguiculata* (XP_027910775).

FIGURE S2 | UV-Visible spectra of MtGlb1-2.1 and MtGlb1-2.4 proteins with HT. (A) WT1, **(B)** 74, **(C)** 238, **(D)** 74/238, **(E)** WT4 and **(F)** 109. For each Glb, the figure shows the spectra of the ferric form (3+), the deoxyferrous form (2+), the complex of ferric globin with cyanide (3+CN), and the complexes of ferrous globin with O_2 (2+O_2), CO (2+CO) and NO (2+NO). The spectra of WT1 and WT4 were obtained with *c.* 18 μM protein in 50 mM potassium buffer (pH 7.0). The spectra of the mutants were obtained with *c.* 18 μM protein in the same buffer supplemented with 150 mM NaCl. *sh,* shoulder.

A MtGlb1-2.1 WT1 (HT)

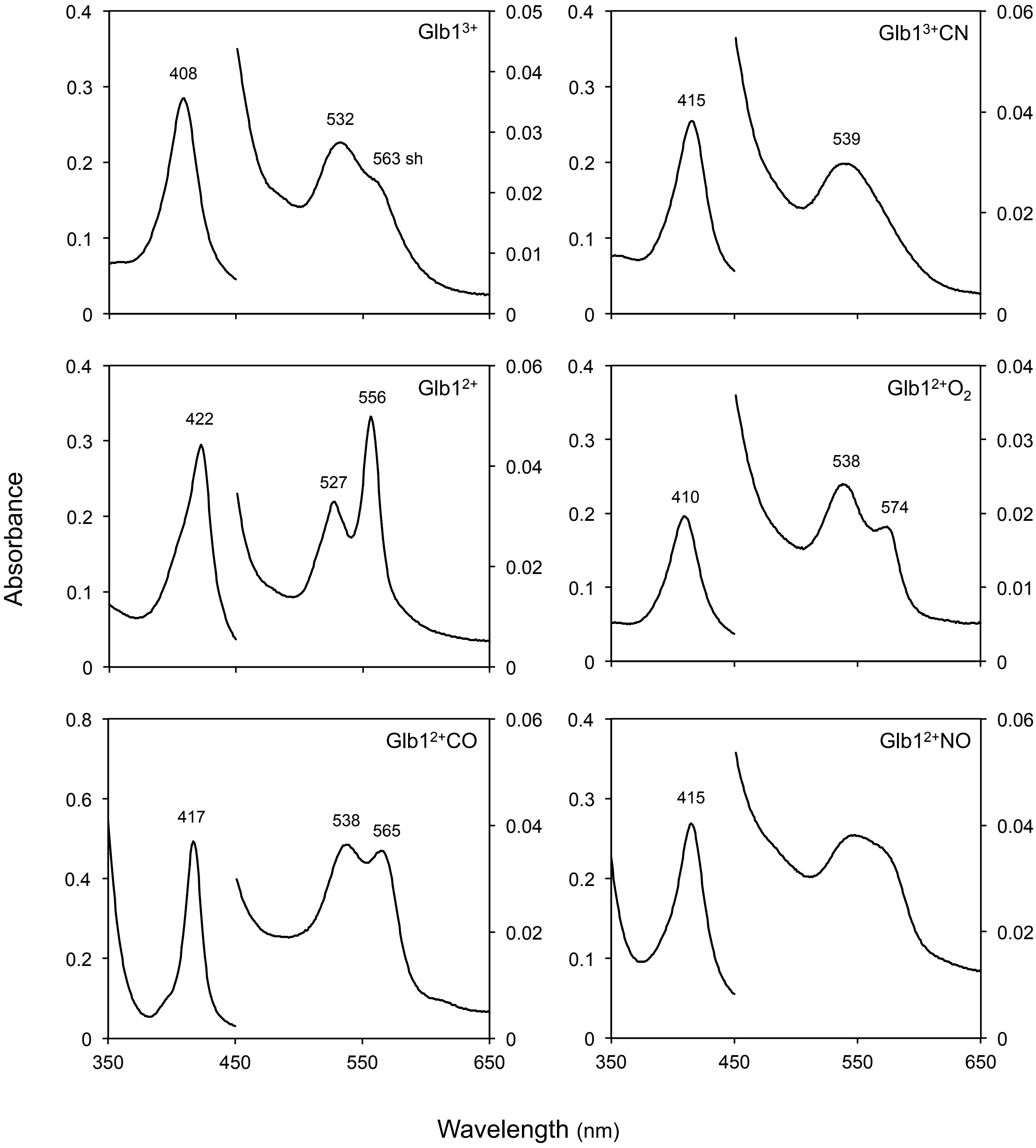

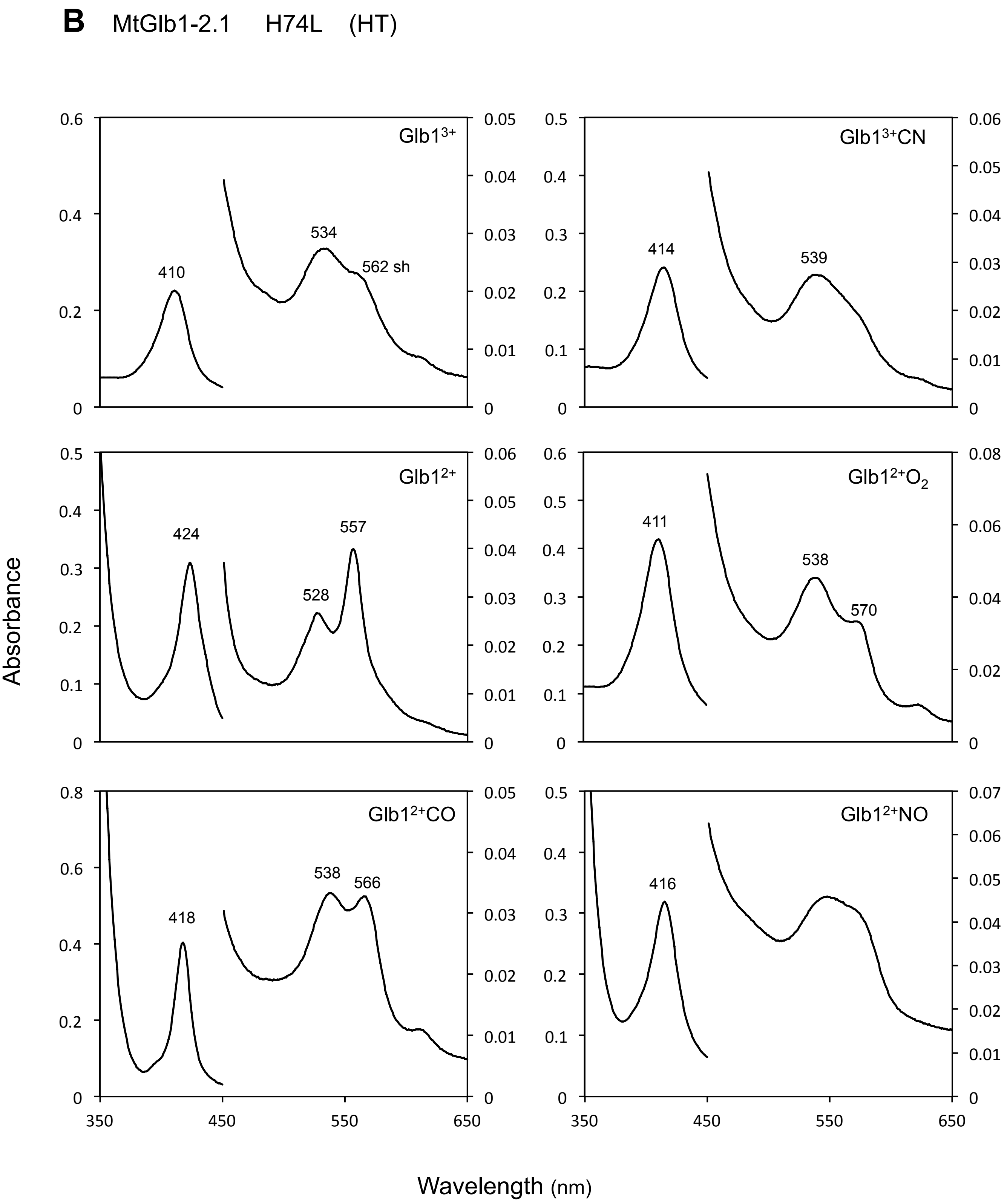
B MtGlb1-2.1 H74L (HT)
Glb1^{3+}
410
534
562 sh
Glb1^{3+}CN
414
539
Glb1^{2+}
424
528
557
Glb1^{2+}O$_2$
411
538
570
Glb1^{2+}CO
418
538
566
Glb1^{2+}NO
416
Absorbance
Wavelength (nm)
350
450
550
650

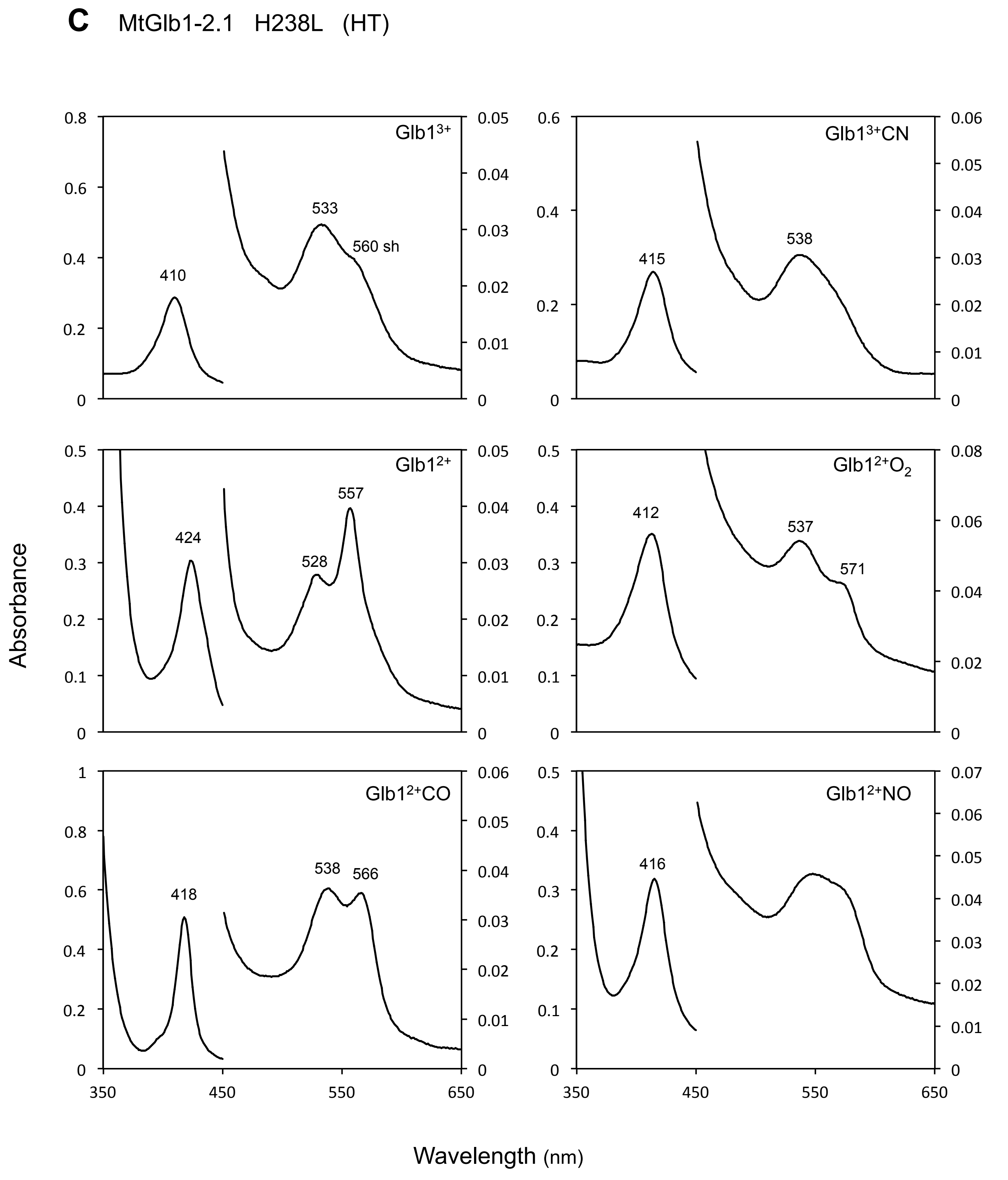
C
MtGlb1-2.1 H238L (HT)
Glb1^{3+}
410
533
560 sh
Glb1^{3+}CN
415
538
Glb1^{2+}
424
528
557
Glb1^{2+}O$_2$
412
537
571
Glb1^{2+}CO
418
538
566
Glb1^{2+}NO
416
Absorbance
Wavelength (nm)
350
450
550
650

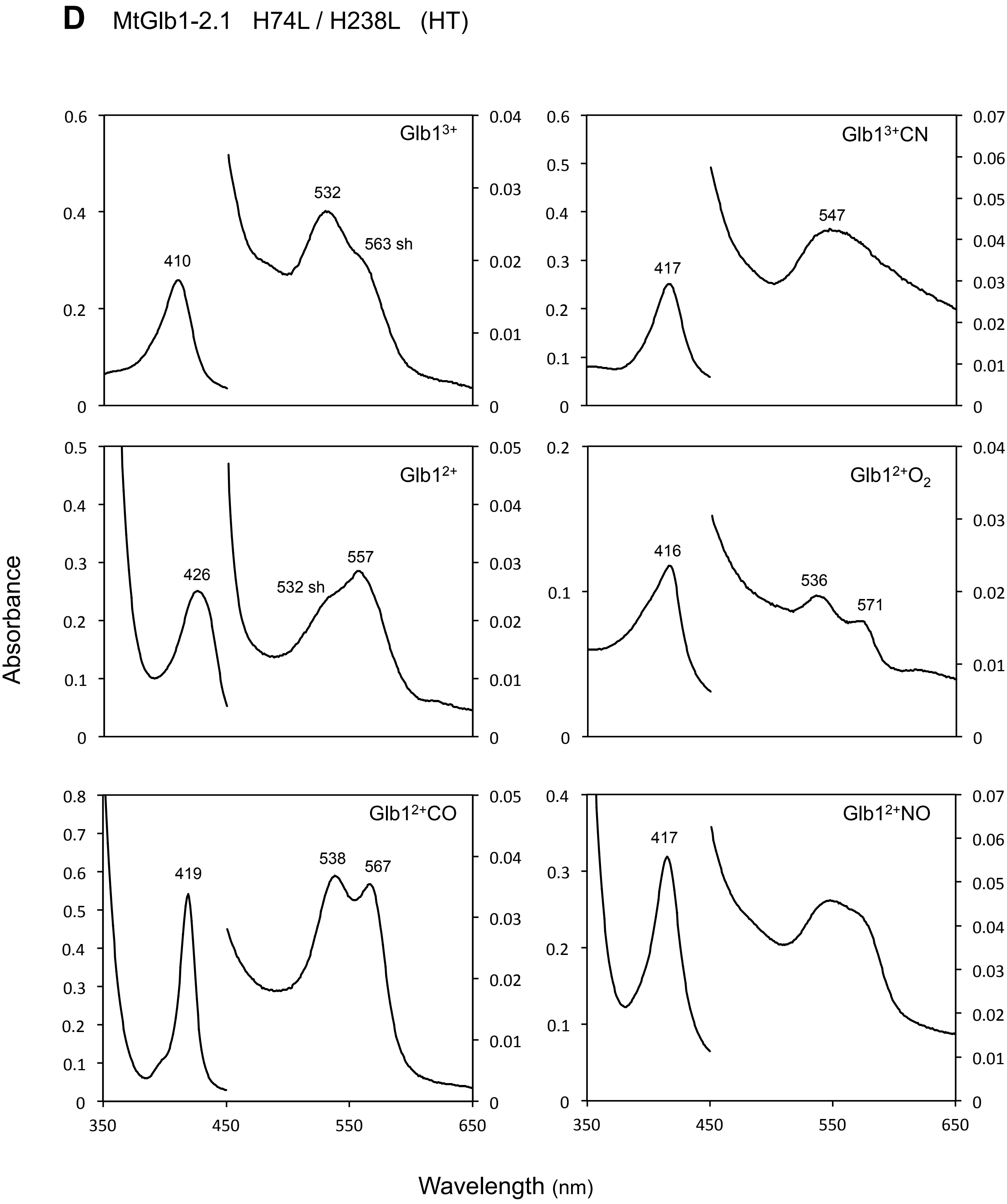

D MtGlb1-2.1 H74L / H238L (HT)
Glb1^{3+}
410
532
563 sh
Glb1^{3+}CN
417
547
Glb1^{2+}
426
532 sh
557
Glb1^{2+}O$_2$
416
536
571
Glb1^{2+}CO
419
538
567
Glb1^{2+}NO
417
Absorbance
Wavelength (nm)
350
450
550
650

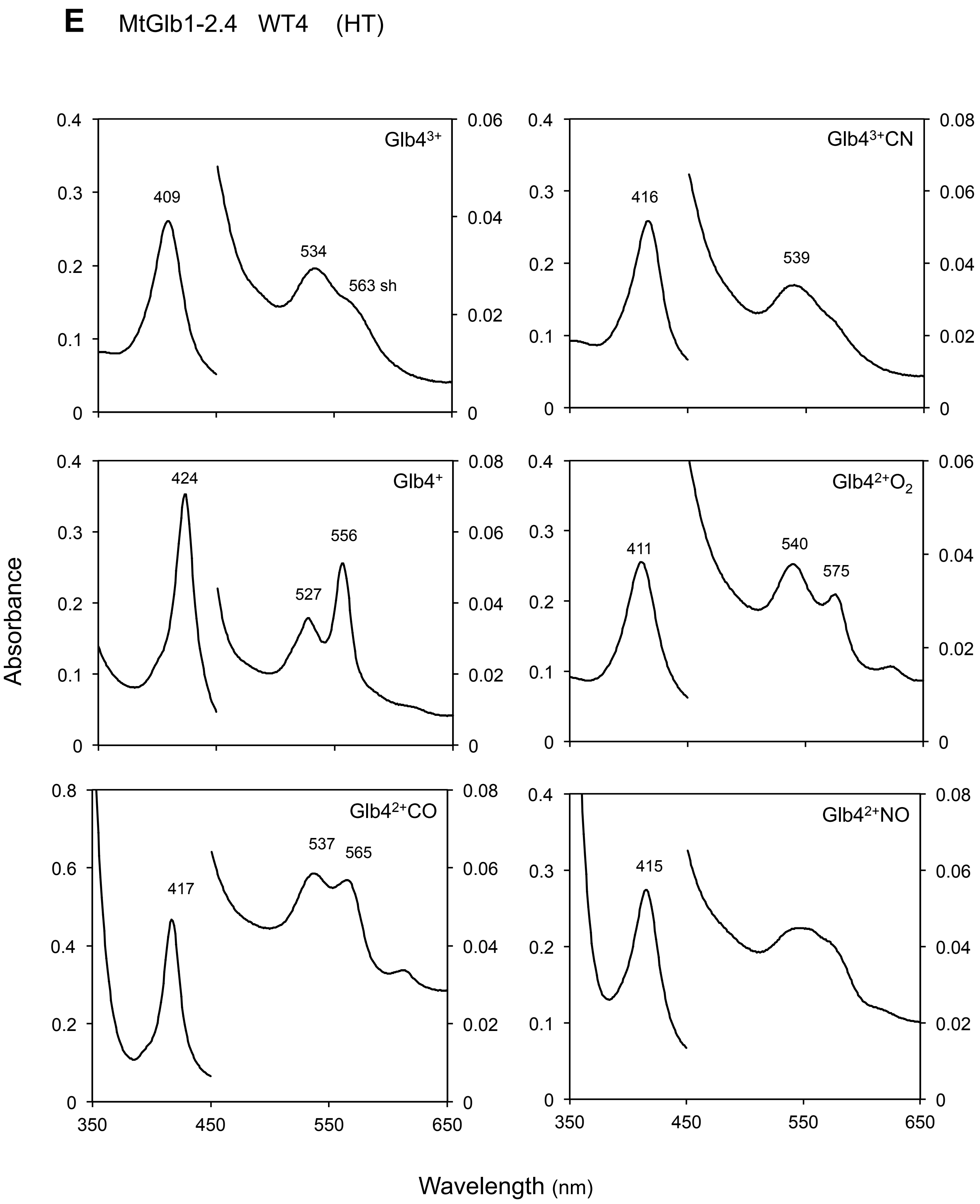
E MtGlb1-2.4 WT4 (HT)
Glb4^{3+}
409
534
563 sh
Glb4^{3+}CN
416
539
Glb4^{+}
424
527
556
Glb4^{2+}O$_2$
411
540
575
Glb4^{2+}CO
417
537
565
Glb4^{2+}NO
415
Absorbance
Wavelength (nm)
350
450
550
650

F MtGlb1-2.4 H109L (HT)

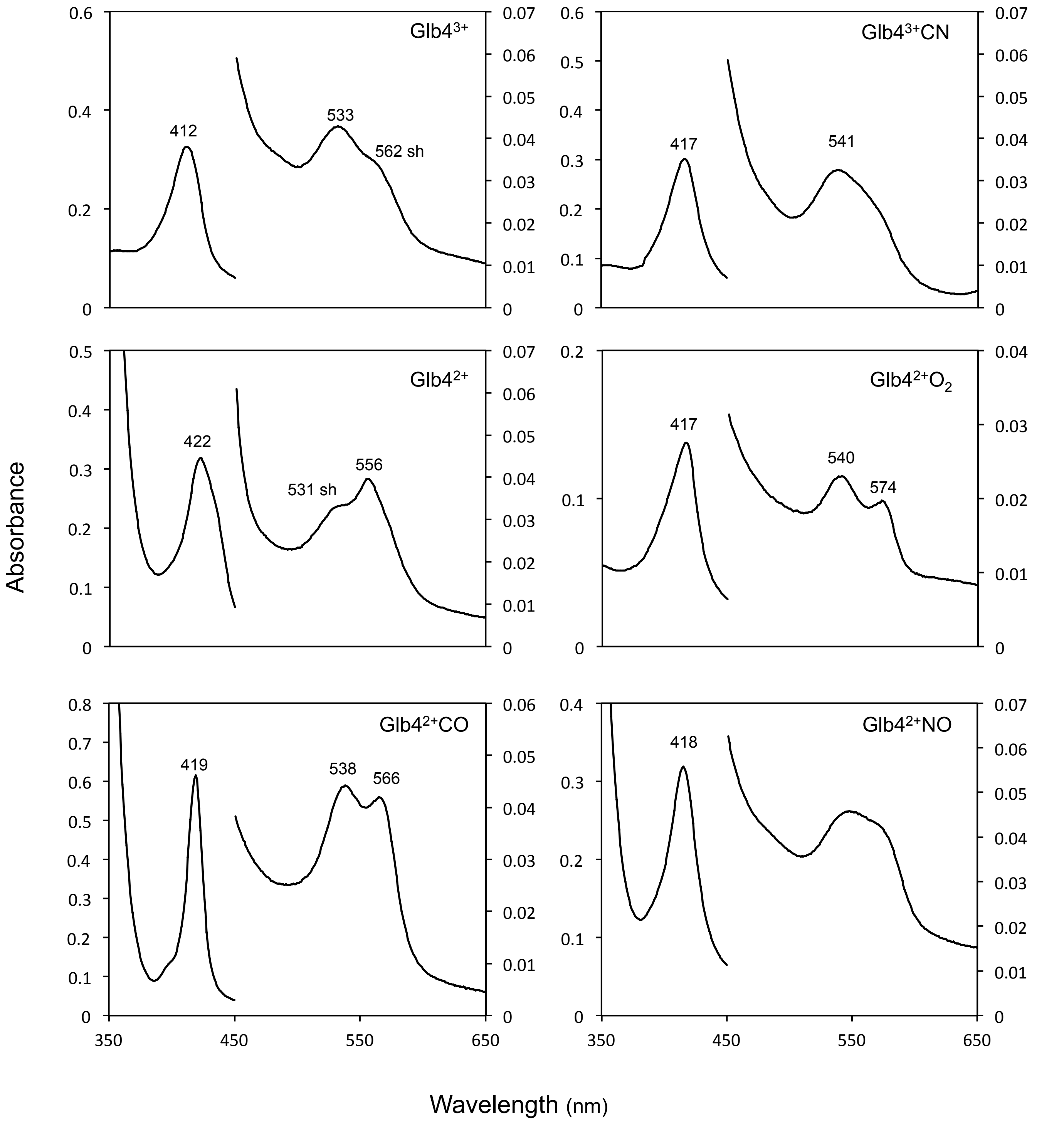

FIGURE S3 | UV-Visible spectra of MtGlb1-2.1 with Strep-tag (ST). (A) WT1, **(B)** 74, **(C)** 238 and **(D)** 74/238. For each globin, the figure shows the spectra of the ferric form (3+), the deoxyferrous form (2+), the complex of Glb^{3+} with cyanide (3+CN), and the complexes of Glb^{2+} with O_2 ($2+O_2$), CO (2+CO) and NO (2+NO). The spectra of WT1 were obtained with *c.* 20 µM globin in 50 mM potassium buffer (pH 7.0). The spectra of the mutants were obtained with *c.* 20 µM globin in the same buffer supplemented with 150 mM NaCl. *sh*, shoulder.

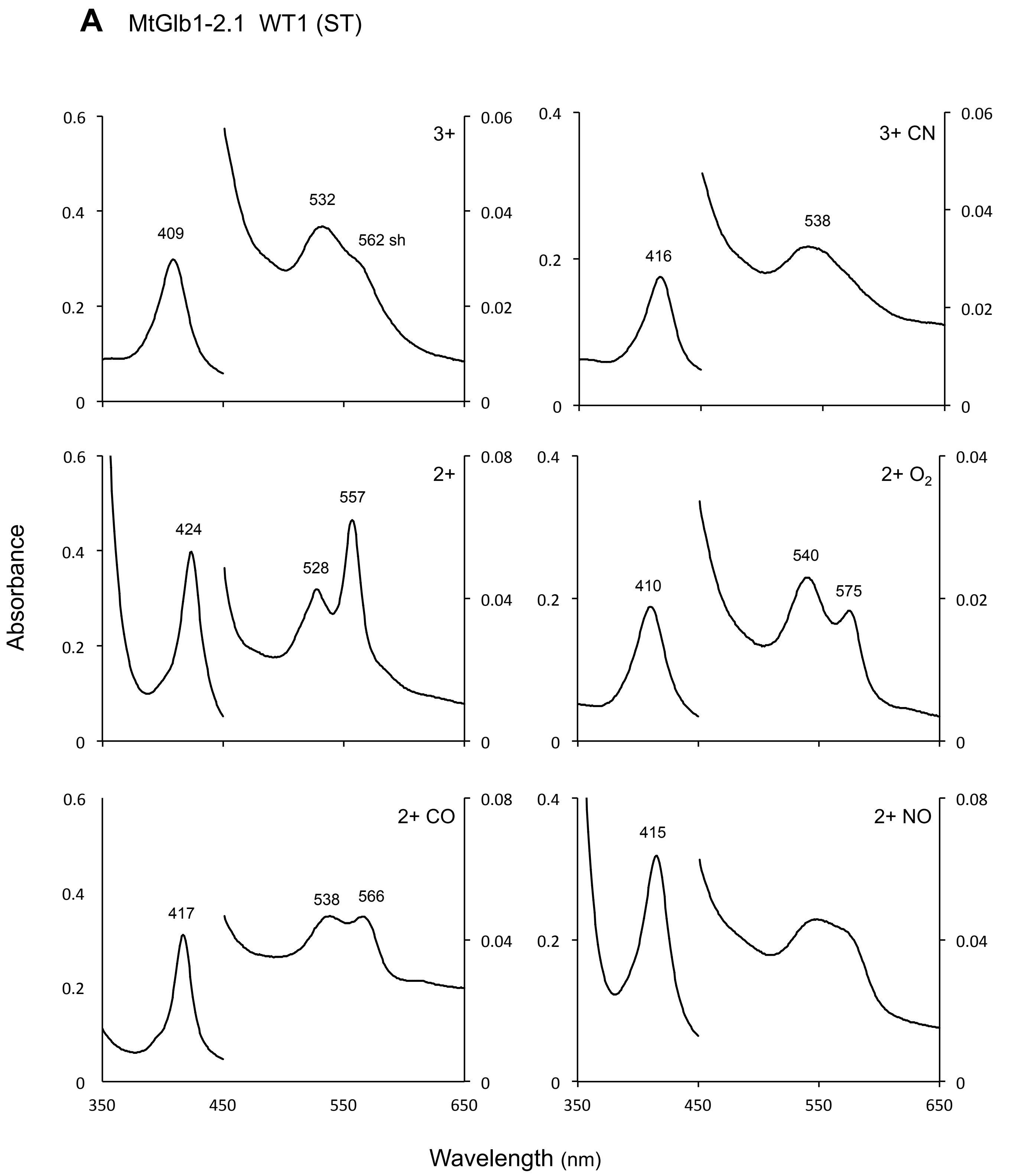
A
MtGlb1-2.1 WT1 (ST)
3+
409
532
562 sh
3+ CN
416
538
2+
424
528
557
2+ O2
410
540
575
2+ CO
417
538
566
2+ NO
415
Absorbance
Wavelength (nm)
350
450
550
650

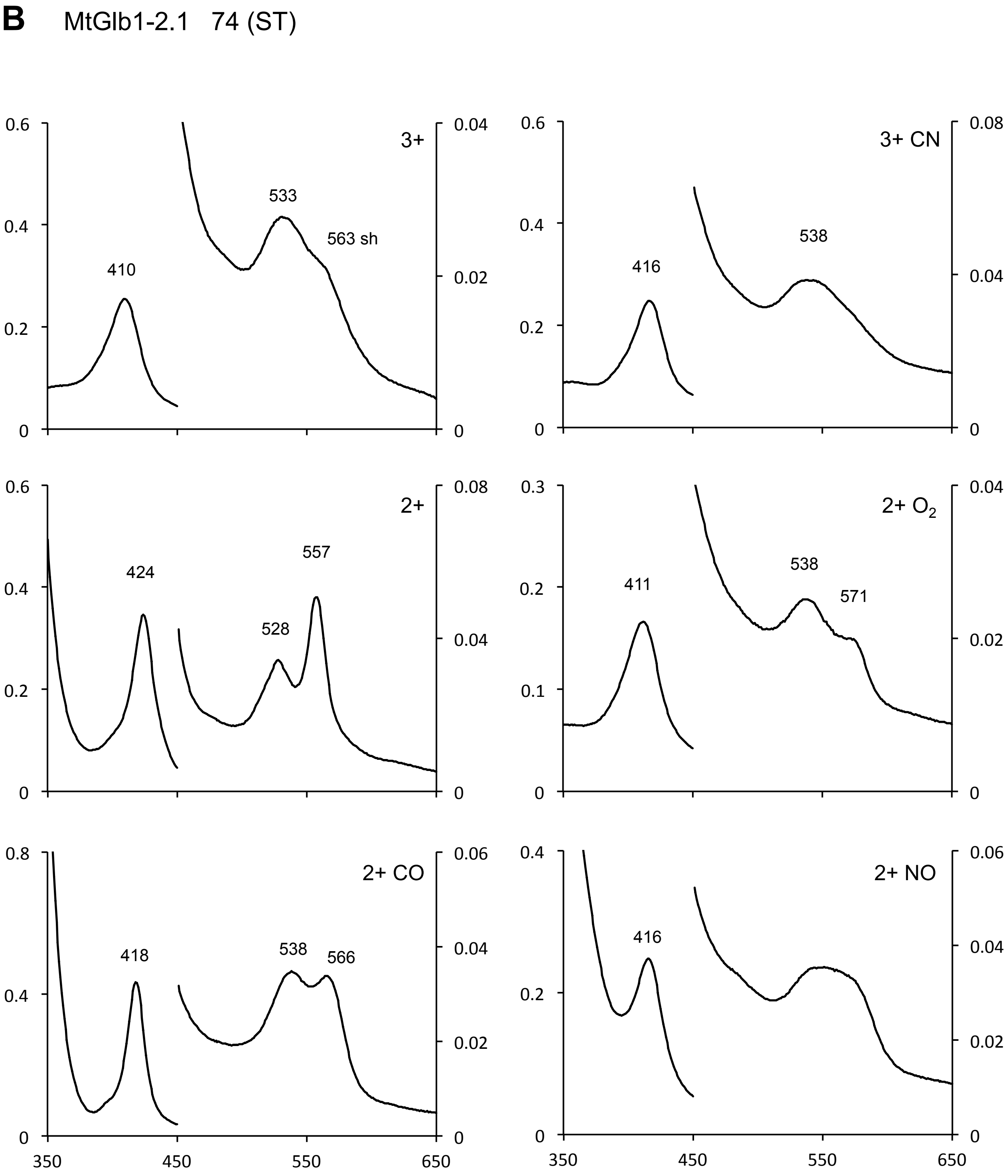

B
MtGlb1-2.1 74 (ST)
3+
410
533
563 sh
3+ CN
416
538
2+
424
528
557
2+ O2
411
538
571
2+ CO
418
538
566
2+ NO
416
538
350
450
550
650

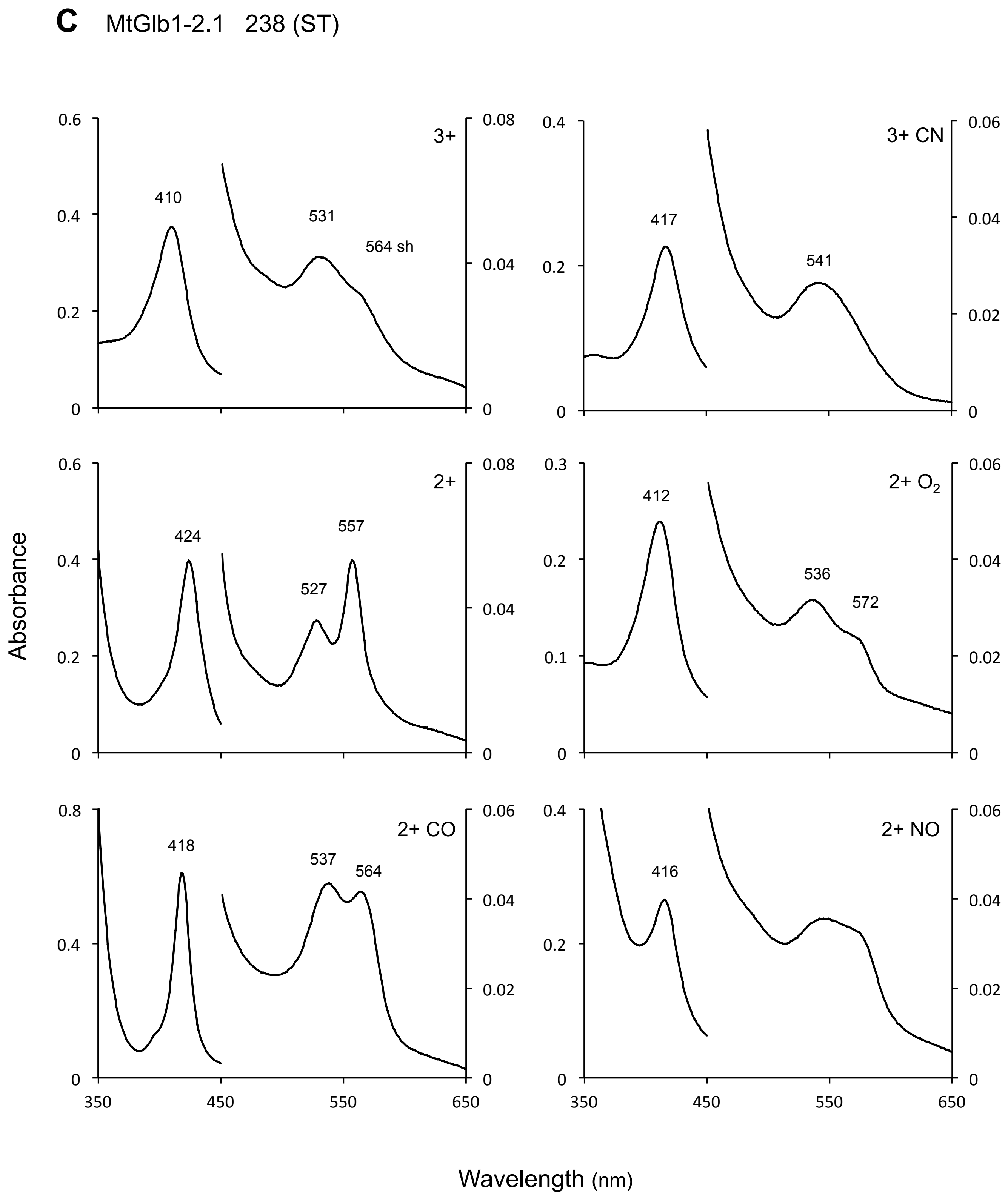

C MtGlb1-2.1 238 (ST)
3+
410
531
564 sh
3+ CN
417
541
2+
424
527
557
2+ O2
412
536
572
2+ CO
418
537
564
2+ NO
416
Absorbance
Wavelength (nm)

D MtGlb1-2.1 74/238 (ST)

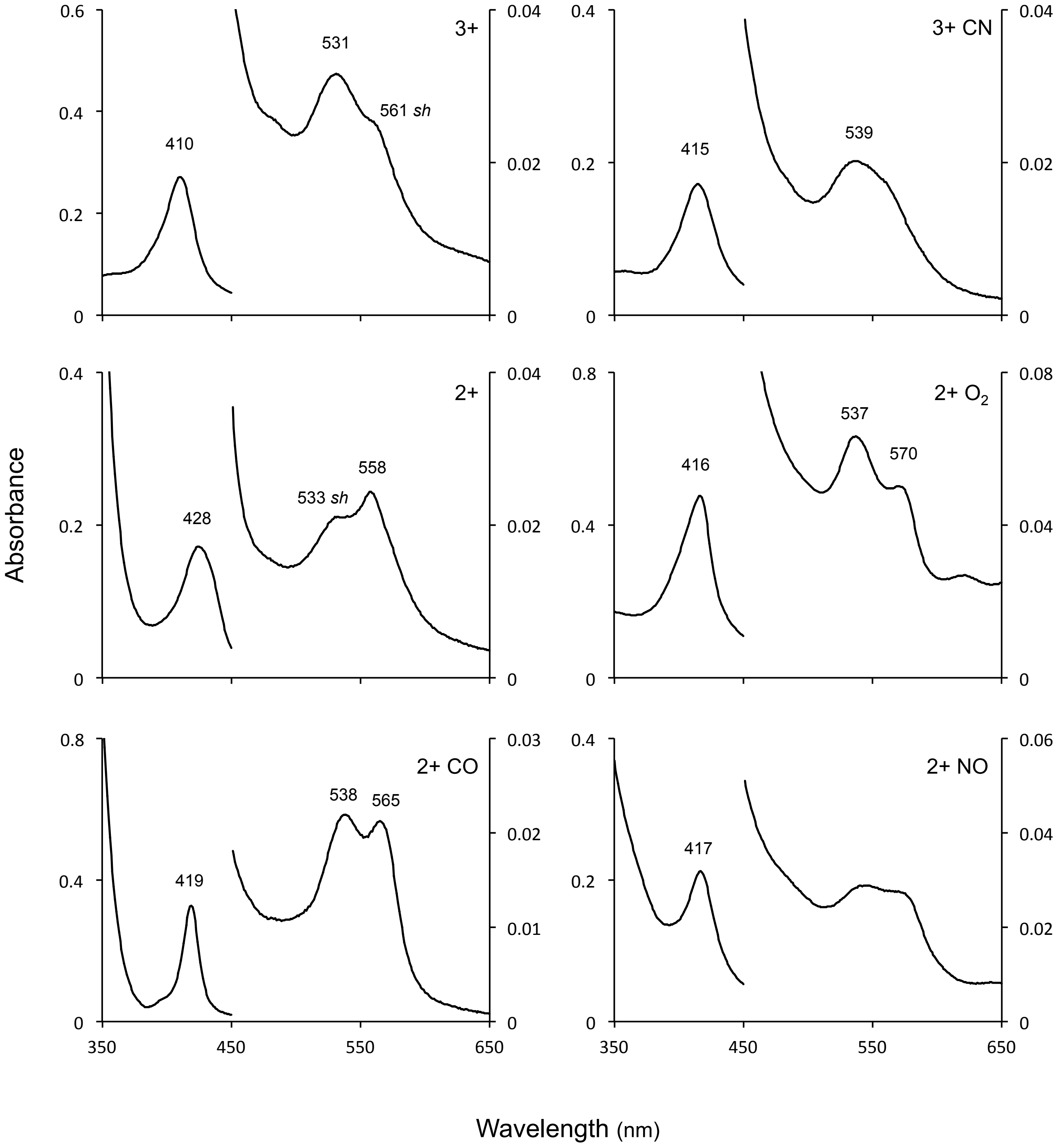

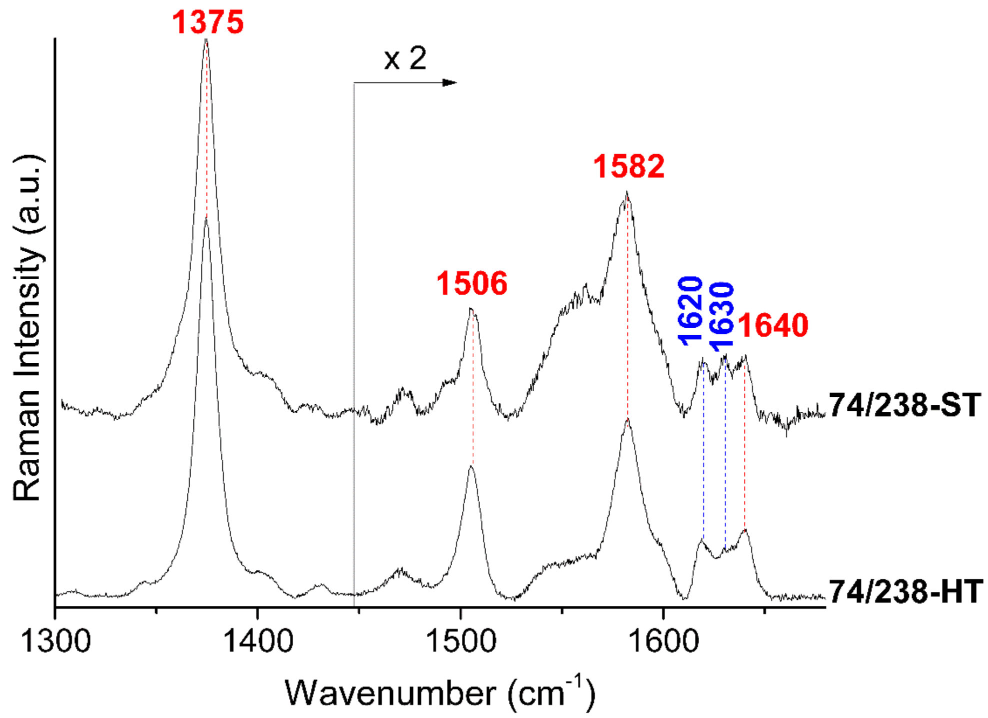

FIGURE S4 | High-frequency region RR spectra of the ferric 74/238 mutant purified with His-tag (HT) and Strep-tag (ST). The spectra have been shifted along the *y*-axis and the 1450–1700 cm^{-1} region has been expanded two-fold for better visualization. Experimental conditions: excitation at 406.7 nm, laser power at the sample 5 mW, average of four spectra with 40 min integration time (74/238 HT) or average of six spectra with 30 min integration time (74/238 ST).

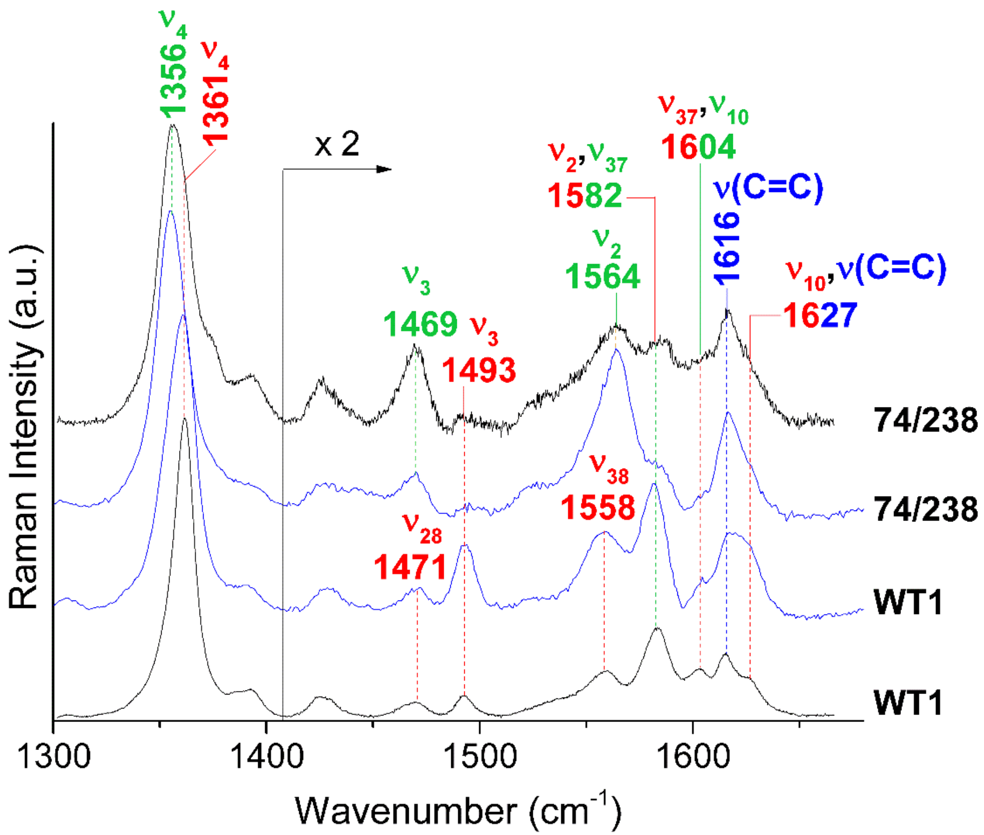

FIGURE S5 | High-frequency region RR spectra of deoxyferrous WT1 and 74/238. The spectra were obtained with excitation at 413.1 nm (black traces), in resonance with the 6cLS species, and excitation at 441.6 nm (blue traces), in resonance with the 5cHS species. The wavenumbers of the core size marker bands of the 6cLS and 5cHS species and the vinyl ν(C=C) stretching modes are reported in red, green, and blue, respectively. The spectra have been shifted along the *y*-xis and the 1430–1700 cm^{-1} region has been expanded two-fold for better visualization. Experimental conditions at 413.1 nm: laser power at the sample 5 mW, average of three spectra with 30 min integration time (all proteins). Experimental conditions at 441.6 nm: laser power at the sample 10 mW, average of four spectra with 12 min integration time (WT1), average of four spectra with 10 min integration time (74/238).

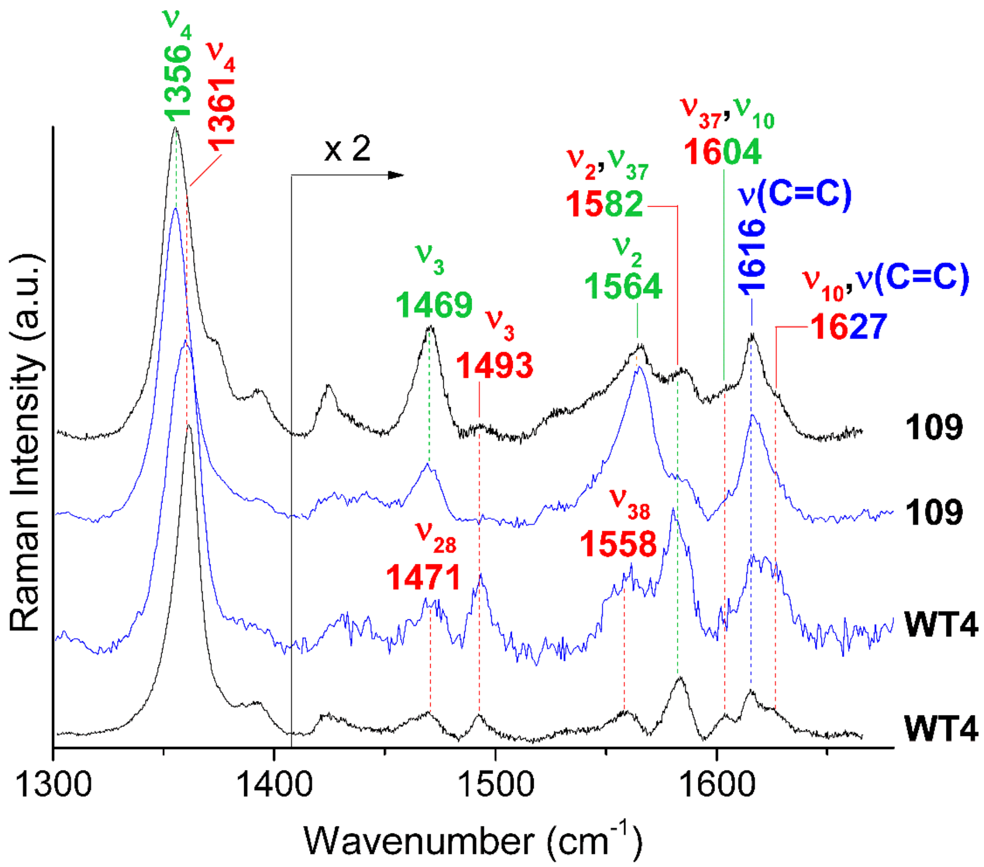

FIGURE S6 | High-frequency region RR spectra of deoxyferrous WT4 and 109. The spectra were obtained with excitation at 413.1 nm (black traces), in resonance with the 6cLS species, and excitation at 441.6 nm (blue traces), in resonance with the 5cHS species. The wavenumbers of the core size marker bands of the 6cLS and 5cHS species and the vinyl ν(C=C) stretching modes are reported in red, green, and blue, respectively. The spectra have been shifted along the *y*-axis and the 1430–1700 cm^{-1} region has been expanded two-fold for better visualization. Experimental conditions at 413.1 nm: laser power at the sample 5 mW, average of three spectra with 30 min integration time (all proteins). Experimental conditions at 441.6 nm: laser power at the sample 10 mW, average of three spectra with 9 min integration time (WT4), average of six spectra with 18 min integration time (109).

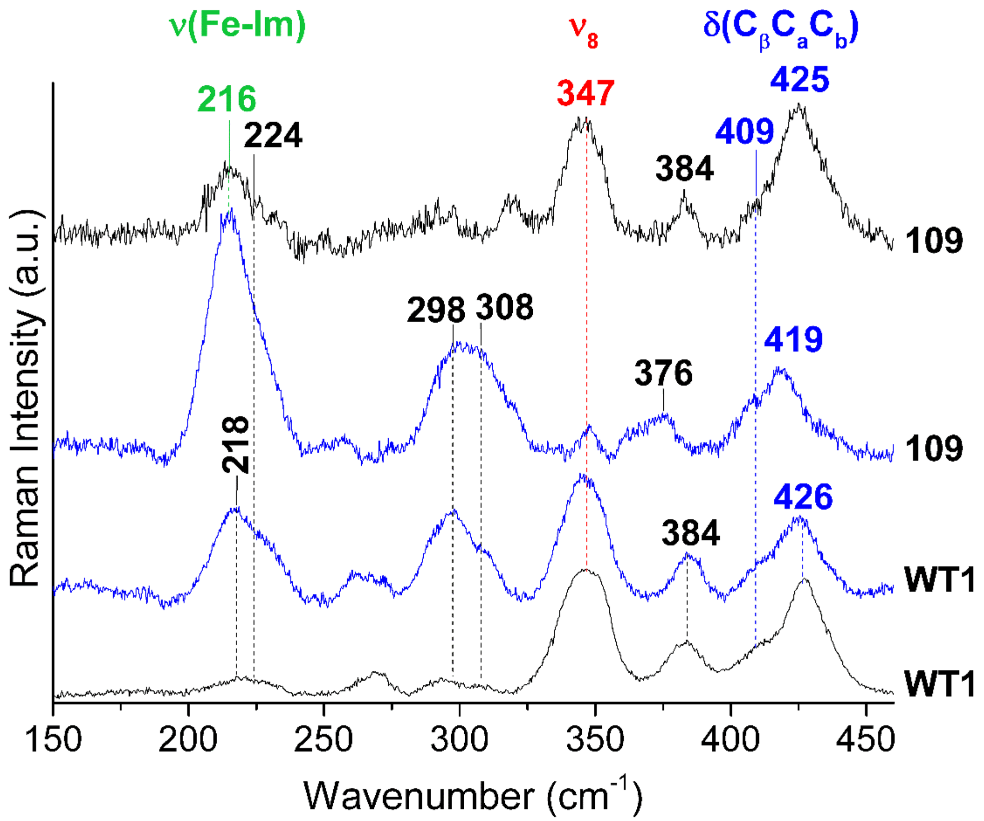

FIGURE S7 | Low-frequency region RR spectra of deoxyferrous WT1 and 109. The spectra were obtained with excitation at 413.1 nm (black traces), in resonance with the 6cLS species, and excitation at 441.6 nm (blue traces), in resonance with the 5cHS species. The wavenumbers of the marker bands of the 6cLS and 5cHS species and the vinyl $\delta(C_{\beta}C_{a}C_{b})$ bending modes are reported in red, green and blue, respectively. The spectra have been shifted along the *y*-axis for better visualization. Experimental conditions with excitation at 413.1 nm: laser power at the sample 5 mW, average of four spectra with 40 min integration time (WT1), average of three spectra with 30 min integration time (109). Experimental conditions with excitation at 441.6 nm: laser power at the sample 10 mW, average of six spectra with 60 min integration time (WT1) and average of three spectra with 30 min integration time (109).

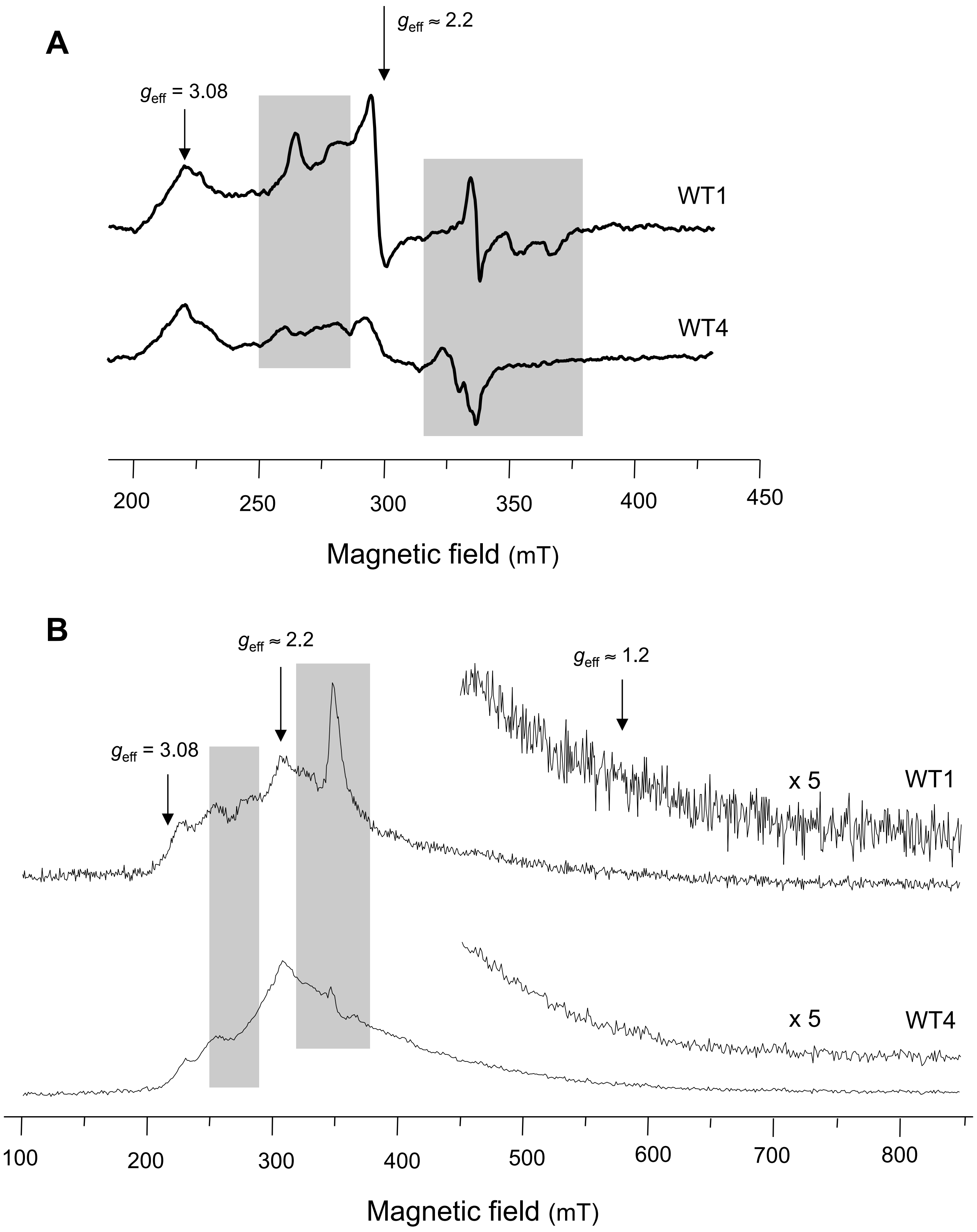

FIGURE S8 | EPR spectra. (A) Continuous-wave (CW) EPR spectra of ferric WT1 and WT4. The spectra were measured at 15 K. Field regions with features corresponding to very minor populations of other low spin and heme-NO forms are shadowed. Positions and the effective *g* (g_{eff}) values of the signals associated to the HALS center are marked (see text). **(B)** 3p ei-EPR spectra of ferric WT1 and WT4. The shape of the spectra is similar to the CW-EPR one in absorption mode. Field regions with features corresponding to very minor populations of other low spin and heme-NO forms are shadowed. Positions and the g_{eff} values of the signals associated to the HALS center are marked. Position corresponding to the lowest g principal value (g_x) cannot be detected in CW-EPR spectra as it would display a very broad feature. It was determined in the ei-EPR spectra by the presence of echo at high field values (see text).

TABLE S1 | RNA-sequencing values of the four splice forms of the *MtGlb1-2* gene in various plant organs.

	MtGlb1-2.1			*MtGlb1-2.2*			*MtGlb1-2.3*			*MtGlb1-2.4*		
	**R1**	**R2**	**R3**	**R1**	**R2**	**R3**	**R1**	**R2**	**R3**	**R1**	**R2**	**R3**
Root	0.51	1.38	2.85	4.49	7.13	7.70	0.11	0.65	1.03	0.00	1.63	0.55
Leaf	0.15	0.00	0.36	1.60	0.00	0.66	0.25	0.00	0.27	0.12	0.00	0.61
Small pod	0.00	0.00	0.00	0.00	0.00	0.00	0.00	0.00	0.00	0.00	0.40	0.00
Medium pod	0.43	0.00	0.00	0.50	0.00	0.03	0.35	0.00	0.04	0.00	0.00	0.00
Large pod	0.00	0.00	0.00	0.35	0.34	0.00	0.39	0.05	0.70	0.00	0.00	0.25
Nod0	0.18	0.00	0.46	1.27	1.07	1.61	1.50	0.28	0.18	0.00	0.00	0.00
Nod4	0.13	0.00	0.00	1.74	0.43	0.00	0.15	0.25	0.39	0.15	0.00	0.00
Nod10	0.00	0.00	0.23	1.71	0.00	0.42	0.05	0.00	0.19	0.00	0.00	0.00
Nod14	0.16	0.00	0.51	0.42	1.45	0.89	0.41	0.16	0.66	0.00	0.14	0.21
Nod28	0.17	0.21	0.00	1.11	0.38	1.00	0.39	0.49	0.24	0.00	0.00	0.16
Nod14 + 12 h	1.21	0.58	0.35	2.78	2.16	1.25	1.21	0.14	0.91	0.64	0.00	0.00
Nod14 + 48 h	0.00	0.49	2.28	1.04	0.61	2.00	0.42	0.30	2.63	0.00	0.15	0.24

Data are three biological replicates (R1 to R3) and correspond to the means and SE's given in Figures 1C and D. Values are expressed in fragments per kilobase of transcript per million reads mapped (FPKM). Nod0 are roots before inoculation; Nod 4 are root bumps at 4 days post-inoculation (dpi); Nod10, Nod14 and Nod28 are nodules at 10, 14 and 28 dpi; and Nod14 + 12 h and Nod14 + 48 h are nodules at 14 dpi that were treated for 12 h and 48 h with 10 mM KNO_3, respectively. Nod, nodules of 4, 10, 18 or 28 days of age.
